# The Natural Product β-Escin Targets Cancer and Stromal Cells of the Tumor Microenvironment to Inhibit Ovarian Cancer Metastasis

**DOI:** 10.3390/cancers13163931

**Published:** 2021-08-04

**Authors:** Hilary A. Kenny, Peter C. Hart, Kasjusz Kordylewicz, Madhu Lal, Min Shen, Betul Kara, Yen-Ju Chen, Niklas Grassl, Yousef Alharbi, Bikash R. Pattnaik, Karen M. Watters, Manish S. Patankar, Marc Ferrer, Ernst Lengyel

**Affiliations:** 1 Department of Obstetrics and Gynecology, Section of Gynecologic Oncology, University of Chicago, Chicago, IL 60637, USA; phart02@roosevelt.edu (P.C.H.); kasjuszk@bsd.uchicago.edu (K.K.); karabetu@msu.edu (B.K.); ychen@vorbiopharma.com (Y.-J.C.); kmwatters@uchicago.edu (K.M.W.); elengyel@uchicago.edu (E.L.); 2 Division of Preclinical Innovation, National Center for Advancing Translational Sciences (NCATS), NIH, Rockville, MD 20852, USA; Madhu.Lal-Nag@fda.hhs.gov (M.L.); shenmin@mail.nih.gov (M.S.); marc.ferrer@nih.gov (M.F.); 3 Department of Proteomics and Signal Transduction, Max Planck Institute of Biochemistry, 82152 Martinsried, Germany; niklasgrassl@gmx.de; 4 Department of Obstetrics and Gynecology, University of Wisconsin-Madison, Madison, WI 53792, USA; yhrby@qu.edu.sa (Y.A.); patankar@wisc.edu (M.S.P.); 5 Department of Pediatrics and Ophthalmology and Visual Sciences, University of Wisconsin-Madison, Madison, WI 53706, USA; pattnaik@wisc.edu

**Keywords:** β-escin, ovarian cancer, tumor microenvironment, metastasis, Digitoxin, Ouabain, high-throughput screening

## Abstract

**Simple Summary:**

β-escin, a component of horse chestnut seed extract, was first identified as an inhibitor of ovarian cancer (OvCa) adhesion/invasion in our high-throughput screening program using a three-dimensional organotypic model assembled from primary human cells and extracellular matrix. The goal of the study presented here is to determine if β-escin and structurally-similar compounds have a therapeutic potential against OvCa metastasis. β-escin and cardiac glycosides inhibit ovarian cancer adhesion/invasion to the omental microenvironment in vivo, and β-escin inhibits ovarian cancer metastasis in the prevention and intervention setting. Additionally, β-escin was found to decrease the stemness of ovarian cancer cells, inhibit extracellular matrix production in the tumor microenvironment, and inhibit HIF1α stability in ovarian cancer cells and the tumor microenvironment. This study reveals that the natural compound β-escin has therapeutic potential because of its ability to prevent OvCa dissemination by targeting both cancer and stromal cells in the OvCa tumor microenvironment.

**Abstract:**

The high mortality of OvCa is caused by the wide dissemination of cancer within the abdominal cavity. OvCa cells metastasize to the peritoneum, which is covered by mesothelial cells, and invade into the underlying stroma, composed of extracellular matrices (ECM) and stromal cells. In a study using a three-dimensional quantitative high-throughput screening platform (3D-qHTS), we found that β-escin, a component of horse chestnut seed extract, inhibited OvCa adhesion/invasion. Here, we determine whether β-escin and structurally similar compounds have a therapeutic potential against OvCa metastasis. Different sources of β-escin and horse chestnut seed extract inhibited OvCa cell adhesion/invasion, both in vitro and in vivo. From a collection of 160 structurally similar compounds to β-escin, we found that cardiac glycosides inhibited OvCa cell adhesion/invasion and proliferation in vitro, and inhibited adhesion/invasion and metastasis in vivo. Mechanistically, β-escin and the cardiac glycosides inhibited ECM production in mesothelial cells and fibroblasts. The oral administration of β-escin inhibited metastasis in both OvCa prevention and intervention mouse models. Specifically, β-escin inhibited ECM production in the omental tumors. Additionally, the production of HIF1α-targeted proteins, lactate dehydrogenase A, and hexokinase 2 in omental tumors was blocked by β-escin. This study reveals that the natural compound β-escin has a therapeutic potential because of its ability to prevent OvCa dissemination by targeting both cancer and stromal cells in the OvCa tumor microenvironment.

## 1. Introduction

A number of very successful cancer treatments have been derived from natural sources [1]. Paclitaxel, for example, was first isolated from the bark of the Pacific yew (*Taxus brevifolia)* in 1962, and was later found to have an anti-tumor activity, leading to FDA approval for ovarian cancer (OvCa) treatment in 1992. To date, paclitaxel, which is currently used as a standard of care for advanced breast, lung, and ovarian cancers, is one of the most efficient cancer drugs ever manufactured, proving the potential of natural compounds for cancer treatment [2].

β-escin, a natural pentacyclic triterpenoid saponin, is the main active component in the seed of the horse chestnut, *Aesculus hippocastanum L.* Horse chestnut seed extract is well known for its anti-edematous, anti-inflammatory, and anti-carcinogenic properties [3,4]. Indeed, β-escin has been used in herbal and folk medicine for a long time. Most commonly, β-escin and the extract of the horse chestnut seed are advertised and prescribed for the treatment of varicose veins [5]. Notably, this extract is widely available over the counter in pill or oil drop form at health food and drug stores.

In a screen of 2420 compounds, we found preliminary evidence that β-escin inhibits OvCa cell adhesion/invasion, leading us to further examine this natural compound [6]. The anticancer activity of β-escin has also been reported in multiple cancers, including lung, breast, hepatocellular, leukemia, pancreatic, renal, bladder, osteosarcoma, and colorectal cancers [4,7,8,9,10,11,12,13,14,15]. The mode of action of β-escin varies considerably with its concentration and length of treatment. In vitro, β-escin most commonly induces apoptosis by inhibiting several transcription factors, including NFκB, in pancreatic, colorectal, renal, and osteosarcoma cancers [4,9,12,13,15]. β-escin has also exhibited pro-apoptotic effects on hepatocellular, bladder, osteosarcoma, and colorectal cancers in vivo [10,11,13,15]. 

Although OvCa is a rare disease, it is one of the leading causes of cancer-associated mortality in women in the United States [16]. This high mortality is due to wide-spread metastasis throughout the peritoneal cavity at the time of diagnosis, short time to recurrence (~2 years), and eventual resistance to standard of care therapy. Recently approved drugs for second-line treatment in women with high-grade serous OvCa, including anti-angiogenic therapies and poly (ADP-ribose) polymerase inhibitors, have resulted in improved progression-free survival (reviewed in [17,18]). However, the cost of these drugs is very high, and to date, they have not been shown to improve overall survival. Moreover, none of the available treatments for OvCa patients specifically target OvCa cell colonization of the peritoneal microenvironment, which is the first step in wide-spread metastasis of the disease. 

Here, we investigate β-escin’s mode of action during OvCa metastasis. Given the existing data on the anti-cancer and anti-inflammatory activity of β-escin, we hypothesized that β-escin and structurally similar compounds act on both cancer and stromal components of the tumor microenvironment and, therefore, could represent an effective treatment for OvCa metastasis. We report that β-escin enhances autophagy, while inhibiting HIF-1α stability and extracellular matrix (ECM) production in the OvCa tumor microenvironment (TME). 

## 2. Results

### 2.1. Activity of β-escin on Ovarian Cancer Cell-Tumor Microenvironment Interactions

**Figure 1:**
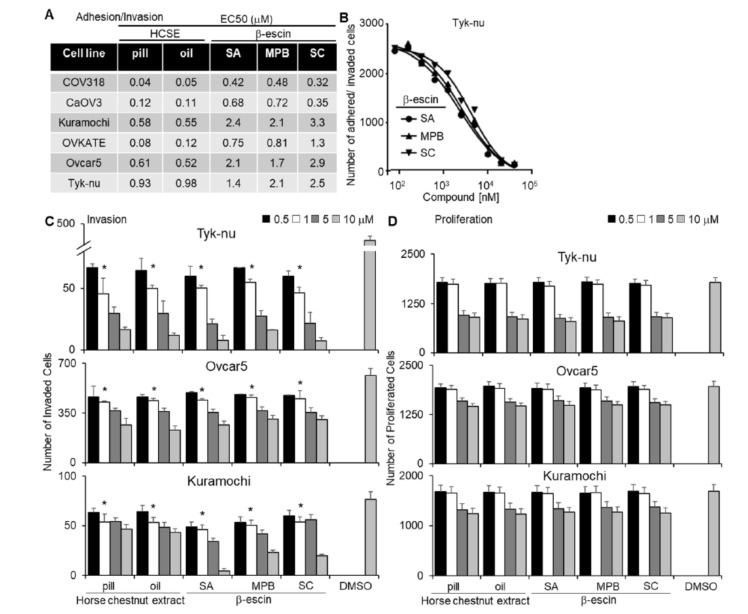
Testing different sources of β-escin in functional assays using a 3D organotypic model. Two horse chestnut extracts (HCSE) and three different sources of β-escin were tested at 12 doses in five high-grade serous ovarian cancer (OvCa) cell lines for their inhibitory effect on OvCa cell adhesion/invasion to the 3D organotypic model (16 h). (**A**) The half-maximal response values (EC_50_) for each β-escin source in each cell line in the adhesion/invasion assays to the 3D organotypic model (16 h). (**B**) Representative dose−response curves of different β-escin sources for the Tyk-nu OvCa cell line in cell adhesion/invasion assays to the 3D organotypic model (16 h, mean ± standard deviation, *n* = 8). (**C**,**D**). Invasion and proliferation assays were performed. The effect of β-escin sources at four doses were tested in three OvCa cell lines. **C**. OvCa cell invasion (24–48 h) was tested using a 96-well Boyden chamber lined with collagen type I and the 3D organotypic culture. (**D**) The effect of β-escin on OvCa cell numbers after treatment (96 h) was tested in 384-well plates on the 3D organotypic culture. Mean ± standard deviation. *, *p* < 0.05, *n* = 5–8. DMSO—dimethyl sulfoxide; SA—Sigma Aldich; MPB—MP Biomedicals; SC—Santa Cruz.

In separate adhesion, invasion, and proliferation assays (Figure 1), we tested purified β-escin from three different sources and horse chestnut extract in both pill form (17% β-escin) and oil solvent form. In a multi-dose 3D adhesion/invasion high-throughput screening (HTS) assay [19], all forms of β-escin and horse chestnut seed extract were observed to inhibit tumor cell adhesion/invasion across all six high-grade serous OvCa cell lines tested (CaOV3, Ovcar4, OVKATE, Kuramochi, Snu-119, and Tyk-nu [20]; Figure 1A,B). The three purified β-escin and the two horse chestnut seed extracts were further evaluated in the three additional functional screens. All β-escin agents inhibited OvCa cell adhesion and invasion to the 3D organotypic culture (fibroblasts, mesothelial cells, fibronectin, and collagen type I [21]; Supplementary Figure S1 and Figure 1C, respectively), but did not significantly affect the number of OvCa cells present on the 3D culture after 4 days (Figure 1D). Moreover, all β-escin agents decreased the ability of OvCa cells to migrate independent of the TME (Supplementary Videos S1–S4).

### 2.2. Identification of Compounds Structurally Similar to β-escin That Block Ovarian Cancer Cell-Tumor Microenvironment Interactions

**Figure 2:**
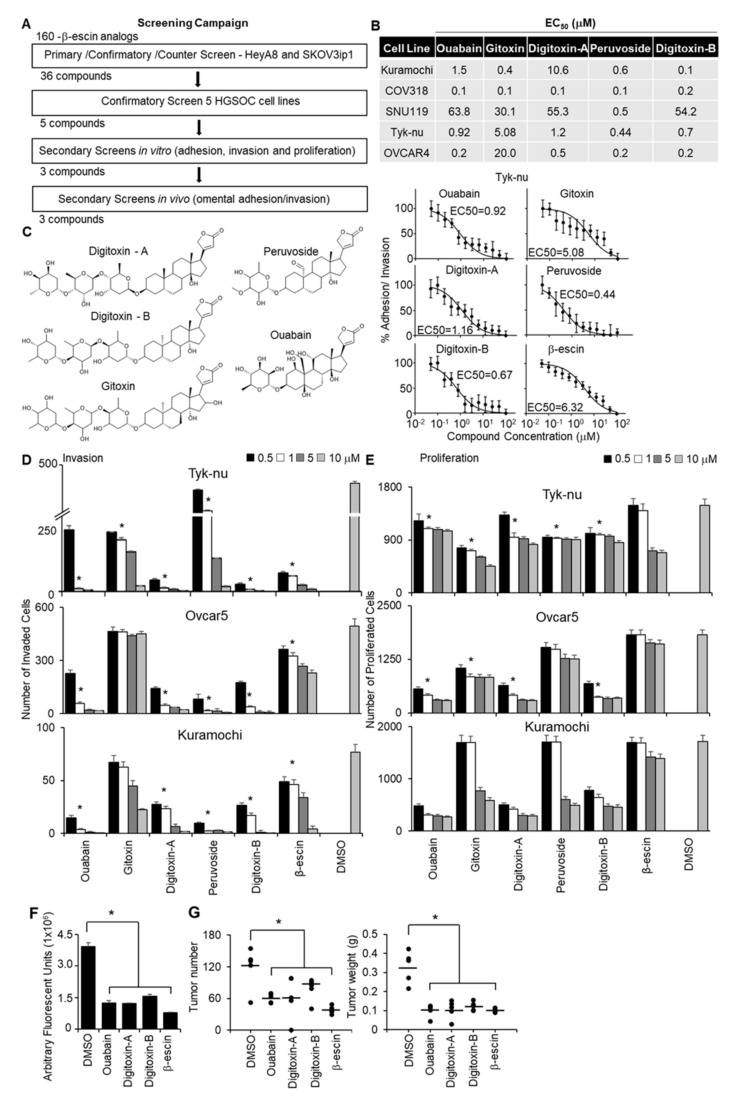
Quantitative high throughput screening with a 3D organotypic model to identify the compounds structurally similar to β-escin that inhibit OvCa metastasis. (**A**) An outline of the screening strategy and the number of active compounds after each assay are shown. (**B**) The half-maximal response values (EC_50_) of each compound for their inhibitory effect on OvCa cell adhesion/invasion to the 3D organotypic model (16 h) are reported. Representative dose−response curves of Tyk-nu OvCa cell adhesion/invasion to the 3D organotypic model (16 h, mean ± standard deviation, *n* = 8). (**C**) Chemical structures of the active compounds. (**D**,**E**) In vivo secondary biological screens. Invasion and proliferation assays. The effect of β-escin sources at four doses were tested in three OvCa cell lines. (**D**) OvCa cancer invasion (24–48 h) was tested using a 96-well Boyden chamber lined with collagen type I as the ECM using the 3D organotypic culture. (**E**) OvCa cell proliferation (96 h) was tested in 384-well plates on the 3D organotypic culture. (**F**,**G**) In vivo biological screens. Adhesion/invasion and prevention metastasis assays were performed. The compounds were tested at a 5 μmol/L dose or dimethyl sulfoxide (DMSO) solvent control (1 μL) in cold phosphate-buffered saline (PBS; 500 μL total). GFP-labeled ID8^p53-/-^ cells (5 million) were mixed with the indicated compound and injected into C57BL/6 mice. (**F**) In vivo adhesion/invasion assay. The mice were sacrificed at 16 h, and the fluorescence signal in the omentum was measured. (**G**) In vivo prevention metastasis assay. After the initial injection of cancer cells and drug (5 μmol/L) or DMSO (1 μL) in PBS (500 μL total), the mice were interperitoneally injected with drug (1 mg/kg/day) or DMSO (1 μL) in PBS (100 μL total) on days 2 and 4. Forty-five days post cancer cell injection, the weight and number of tumors were determined. Mean ± standard deviation. *, *p* < 0.05, *n* = 5–8. DMSO—dimethyl sulfoxide.

A structural similarity search using a 2D Tanimoto coefficient was used to pull out 160 compounds that had ≥ 65% structural similarity (Tanimoto coefficient ≥ 0.65) to β-escin in order to cover a diverse compound set for the follow-up studies. The 160 structurally similar compounds of β-escin were selected from the screening libraries housed at the National Center for Advancing Translational Sciences (NCATS). The screening approach to test these compounds, in terms of inhibiting OvCa metastasis, is shown in Figure 2A. To screen the different β-escin structurally similar compounds, the 3D adhesion/invasion assay was implemented onto a fully automated robotic platform, as previously published [19]. The quality of the HTS assay was robust and reproducible, with signal-to-background ratios of 22- to 63-fold and Z’-factor values of 0.4 to 0.8. The primary screen identified 54 compounds that inhibited the adhesion/invasion of SKOV3ip1 cells. A counter screen was performed to identify and eliminate the compounds that were cytotoxic to the OvCa or stromal cells at similar doses within 16 h [19]. In a confirmatory assay, the non-cytotoxic compounds were re-tested in SKOV3ip1 and HeyA8 OvCa cells using 11-doses in the 3D adhesion/invasion HTS assay. Thirty-six compounds were observed to have inhibitory dose-response activity in at least one of the OvCa cell lines. Representative dose-response curves of five compounds active in HeyA8 cells are shown (Supplementary Figure S2A,B). The activity of the 36 compounds was further investigated in a multi-dose combined adhesion/invasion assay using five serous OvCa cell lines (CaOV3, Ovcar4, Kuramochi, Snu-119, and Tyk-nu). Five of the 36 compounds were found to be active in all five serous OvCa cell lines tested (Figure 2B).

The structures of these five compounds, Digitoxin-A, Digitoxin-B (stereochemically different), Peruovoside, Gitoxin, and Ouabain, which are all cardiac glycosides, are shown in Figure 2C. The functional activity of these five compounds was further tested at four doses in three additional functional screens using three serous OvCa cell lines, Ovcar5, Tyk-nu, and Kuramochi (Figure 2D,E; Supplementary Figure S2C). Compounds were considered active in the functional assays if they inhibited adhesion, invasion, or proliferation in two of the three cell lines at a concentration of 1 μmol/L. Three compounds (Ouabain, Digitoxin-A, and Digitoxin-B) inhibited adhesion to the 3D organotypic culture after 1 h (Supplementary Figure S2C). Four compounds (Ouabain, Digitoxin-A, Digitoxin-B, and Gitoxin) inhibited OvCa cell invasion through the 3D organotypic culture after 24 h (Figure 2D). Finally, four compounds (Ouabain, Digitoxin-A, Digitoxin-B, and Peruvoside) decreased OvCa cell growth after 72 h when cocultured with the 3D organotypic culture (Figure 2E). The effect of Digitoxin-B on cancer cell migration was evaluated in a wound healing assay in two serous OvCa cell lines, Ovcar5 and Kuramochi (Supplementary Videos S1, S2, S5, and S6). Digitoxin-B inhibited the migration of both OvCa cell lines.

The effect of early treatment (prevention study) was tested using two in vivo assays. First, we performed a short-term adhesion/invasion assay in the mouse peritoneal cavity [19], using the same 16-h time point used in the in vitro studies. ID8^p53-/-^GFP OvCa cells, which were used in a syngeneic mouse model [19,20], were mixed with each of the three most efficient compounds (Ouabain, Digitoxin-A, and Digitoxin-B) or β-escin, immediately injected intraperitoneally into mice, and cancer cell adhesion/invasion was measured 16 h later. Consistent with the in vitro studies, β-escin, Ouabain, Digitoxin-A, and Digitoxin-B inhibited adhesion/invasion to the omentum in vivo (Figure 2F). These agents were subsequently evaluated using a metastasis prevention assay in which OvCa cells were mixed with treatment and immediately injected intraperitoneally. Drug treatment was repeated at days 2 and 4. Following observation for 45 days without intervention, all four compounds were found to reduce the tumor number by at least 40% and the tumor weight by 61% (Figure 2G).

### 2.3. β-escin Targets Stem Cells and Reduces HIF1α Stability

**Figure 3:**
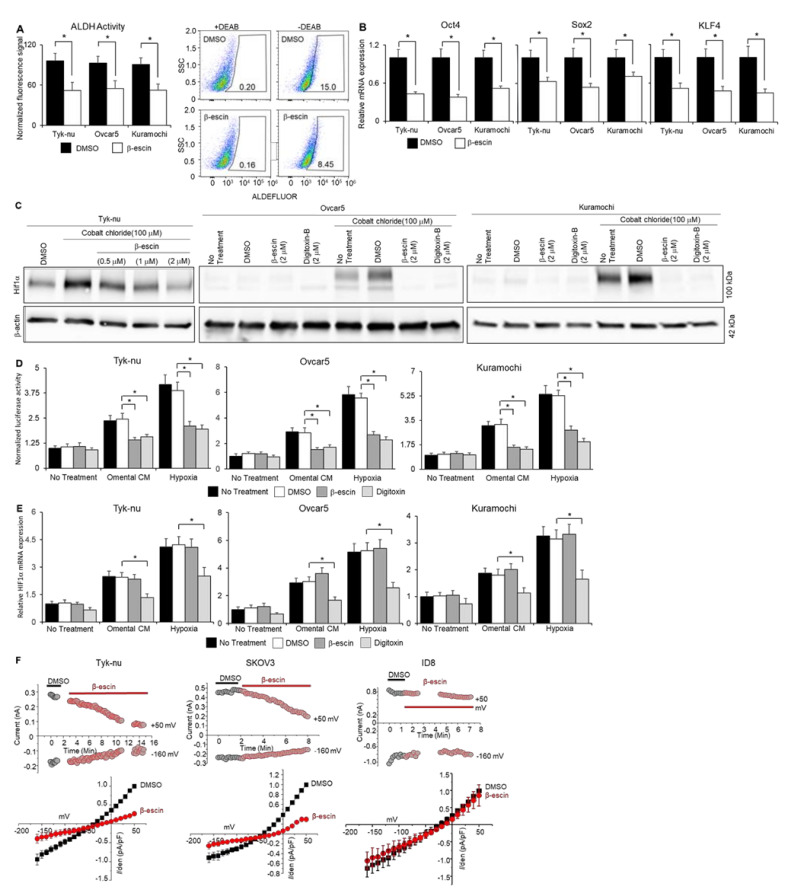
β-escin inhibits ALDH activity, stem cell marker expression, and HIF1α stability in ovarian cancer cells and sodium−potassium pump currents. (**A**) The aldehyde-dehydrogenase (ALDH) activity was measured by flow cytometry in human OvCa cell spheroids after β-escin (10 μM) treatment for 48 h with the ALDEFLUOR^TM^ assay kit (mean ± standard deviation, *n* = 5). Representative images of the flow cytometry analysis of the ALDEFLUOR signal (FITC-Area) in Kuramochi OvCa spheroids. SSC—side scatter-height. * *p* < 0.05. (**B**) Stem cell markers; Sox2, Oct4, and KLF4 mRNA expression were measured by qRT-PCR in human OvCa cell spheroids after β-escin (10 μM) and Digitoxin (0.002 μM) treatment for 48 h. Mean ± standard deviation. *, *n* = 5–8. DMSO—dimethyl sulfoxide. (**C**) HIF1α protein expression was measured by immunoblot analysis in human OvCa cells after β-escin, Digitoxin, or control treatment in combination or not with cobalt chloride treatment for 48 h. (**D**) ODD-luciferase reporter activity in OvCa cells after omental conditioned media (CM; used to partially mimic the omental microenvironment’s secreted factors), 1% oxygen chamber (hypoxia), or no treatment in combination or not with β-escin (10 μM), Digitoxin (0.002 μM), DMSO, or no treatment for 8 h. Mean ± standard deviation. *, *n* = 5. DMSO—dimethyl sulfoxide. (**E**) HIF1α mRNA expression was measured by qRT-PCR in human OvCa cell spheroids after omental CM, hypoxia, or no treatment in combination or not with β-escin (10 μM), Digitoxin (0.002 μM), DMSO, or no treatment for 48 h. Mean ± standard deviation. *, *n* = 5. (**F**) Current traces were measured when the voltage pulse was applied at 50 mV (as outward current) and -160 mV (as inward current) from a holding potential of -60 mV. Top panels, representative time-course experiment shows current measurements in β-escin treated human ovarian cancer (OvCa) cell lines, Tyk-nu (2 µM) and SKOV-3 (10 µM), or the mouse OvCa cell line, ID8 cells (20 µM). Bottom panels, the average normalized current−voltage curves after the application of β-escin (10 μM, grey circle) are shown (*n* = 4). DMSO—dimethyl sulfoxide. DMSO—dimethyl sulfoxide.

Cardiac glycosides function by reducing stem cell populations, inhibiting HIF1α expression, enhancing autophagy, and inhibiting ATP-dependent sodium−potassium exchange across cell membranes [22]. Given that the structurally similar compounds of β-escin identified by our screen were all cardiac glycosides, we investigated whether β-escin acted on OvCa via known cardiac glycoside mechanisms of action, using Digitoxin as the representative cardiac glycoside. To determine the effect of β-escin on the stem cell population in OvCa cell lines, the following four assays were performed: aldehyde dehydrogenase (ALDH) activity assay, real-time PCR analysis of stem cell transcription factors, colony formation assays, and analysis of the production and stability of HIF1α. Treatment with β-escin reduced the ALDH activity (Figure 3A) and decreased the expression of Sox2, Oct4, and KLF4 (Figure 3B) in OvCa cell line spheroids. In addition, β-escin and Digitoxin inhibited colony formation (Supplementary Figure S3), HIF1α protein levels (Figure 3C), and HIF1α stability (Figure 3D), but only Digitoxin inhibited HIF1α mRNA production (Figure 3E). β-escin induced a flux in the autophagy factors ubiquitin-binding protein LC3A/B and SQSTM1/p62 (Supplemental Figure S4). SQSTM1/p62 levels were reduced after 2 h of β-escin treatment in OvCa cell lines (Supplemental Figure S4A), and were recovered 8 h after treatment. In addition, the ratio of LC3A/B decreased within 2–8 h after β-escin treatment in OvCa cell lines. β-escin-mediated autophagy was further confirmed qualitatively using CYTO-ID staining [23]. β-escin and Digitoxin induced CYTO-ID staining, which selectively stained autophagocytic vesicles (Supplemental Figure S4B). Patch-clamp experiments revealed that β-escin inhibits both outward and inward sodium−potassium channel currents in human OvCa cells, but not in the mouse ID8 OvCa cell line (Figure 3F).

### 2.4. β-escin Targets Extracellular Matrix Production in the Omental Microenvironment

**Figure 4:**
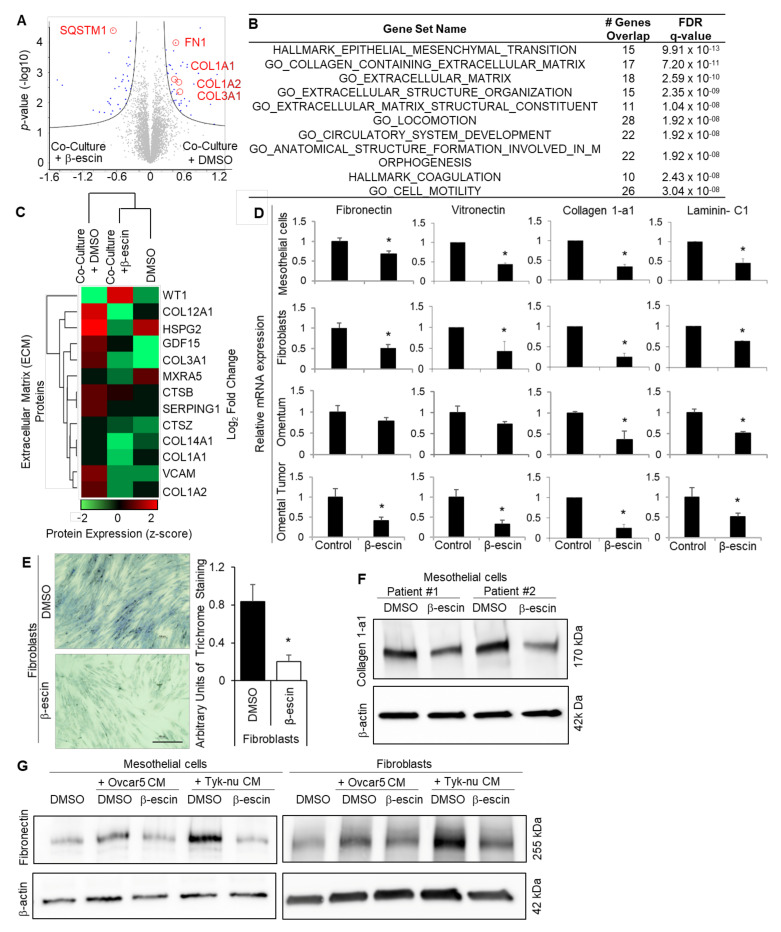
β-escin targets the extracellular matrix production in mesothelial cells. (**A**–**C**) Proteomics. Mass spectrometry analysis of the proteins extracted from the primary human mesothelial cells co-cultured with Tyk-nu OvCa cells and treated with DMSO (control) or β-escin (5 μM) for 48 h. (**A**) Cell lysates were collected and measured on a Q Exactive HF mass spectrometer. Significantly altered proteins following exposure to Tyk-nu co-culture and DMSO or β-escin treatment (5 μM) for 48 h (Volcano plot, Perseus), with the significantly altered proteins highlighted in blue. Significance was defined by an FDR of 0.05 and an S_0_ value of 0.1 (*n* = 4 patients per group in triplicate). The extracellular matrix proteins are defined and circled in red. (**B**) Gene set enrichment analysis (GSEA) of the proteins significantly altered by β-escin in mesothelial cells co-cultured with Tyk-nu cells. “Hallmark” and “Gene Ontology” datasets were included in the analysis. (**C**) Unsupervised hierarchical clustering (Perseus) was performed on the dataset from (**A**), assessing the extracellular matrix-related proteins. (**D**) The fibronectin, vitronectin, collagen-1a1, and laminin-C1 mRNA expressions were measured by qRT-PCR in primary human mesothelial cells, primary human fibroblasts, andex vivo human omentum culture or ex vivo human omental tumor culture after DMSO (control) or β-escin (5 μM) treatment for 36 h (mean ± standard deviation; *, *p* < 0.05). (**E**) Collagen fibers were stained with trichrome stain (blue) in primary human fibroblasts after DMSO (control) or β-escin (5 μM) treatment for 36 h (mean ± standard deviation. *, *p* < 0.05). (**F**) Collagen1-a1 immunoblot analysis of primary human mesothelial cells after DMSO (conrol) or β-escin treatment (5 μM) for 48 h. (**G**) Fibronectin immunoblot analysis of primary human mesothelial cells and primary human fiboroblasts after no treatment (control) or OvCa cell line conditioned media (CM) treatment in combination with or without DMSO (control) or β-escin treatment (5 μM) for 48 h.

Mesothelial cells cover the entire surface of the peritoneal and the pleural cavity, and are, therefore, the first cell type encountered by metastasizing OvCa cells [8]. To obtain a global understanding of the impact of β-escin on the omental microenvironment, which is also covered by a single layer of mesothelial cells [21], we performed unbiased proteomic profiling of β-escin treated human omental mesothelial cells co-cultured with OvCa cells ((Supplementary Figure S5A) [22]. Mass spectrometry analysis revealed that the expression of 123 proteins were altered in theβ-escin treated mesothelial cells, and the expression of 19 proteins significantly changed in the untreated mesothelial cells, whether or not they were co-cultured with OvCa cells (Figure 4A, Supplementary Figure S5B, and Supplementary Tables S1 and S3). Significantly regulated proteins by β-escin in the mesothelial cells included the autophagy factor ubiquitin-binding protein, p62/SQSMT1; HMOX1, which is found upstream or downstream of HIF1α signaling; PTPN13, an ECM degrading protein; and perlecan/HSPG2, which is found in the ECM (Supplementary Tables S1 and S3, and Supplementary Figure S5B). Enrichment analysis of the significantly altered proteins in co-cultured mesothelial cells revealed that β-escin repressed the pathways involved in metastatic progression, including ECM production and epithelial-to-mesenchymal (EMT) transition (Figure 4B, Supplementary Table S2). In addition, unsupervised hierarchical clustering confirmed that β-escin prevented the induction of multiple ECM proteins, including several collagens, in mesothelial cells co-cultured with OvCa cells (Figure 4C). As ECM production plays a crucial role in OvCa metastasis [24,25,26,27,28], we focused on the effect of β-escin on ECM production in the tumor microenvironment. Real-time PCR analysis confirmed that β-escin decreased the mRNA production of several ECM molecules, including fibronectin, vitronectin, collagen-1a1, and laminin-C1, in cultured primary human mesothelial cells, primary human fibroblasts, human normal omentum explants, and human omental tumor explants (Figure 4D). Trichrome staining verified that β-escin inhibited collagen fiber production in primary human fibroblasts (Figure 4E), while the immunoblot analysis showed that β-escin inhibited collagen 1-a1 production in primary human mesothelial cells. Furthermore, immunoblot analysis demonstrated that OvCa cell conditioned media (CM) increased fibronectin production in the mesothelial cells and fibroblasts, and that this increase in fibronectin was prevented by β-escin (Figure 4D). Next, we explored the β-escin regulation of HIF1α in the mesothelial cells, which we have shown previously [29]. Immunoblot analysis shows that cobalt chloride increases the HIF1α protein levels in the mesothelial cells, and that this increase in HIF1α was absent in the cells treated with β-escin or Digitoxin (Supplementary Figure S6A). This decrease in HIF1a protein was determined to be the effect of β-escin on HIF1a stability and not on its production (Figure 3C–E). Other proteins regulated by β-escin in the mesothelial cells (Supplementary Tables S1 and S2), and similarly regulated by β-escin and cardiac glycosides in cancer cells, are SQSMT1/p62 and the ratio of LC3A/B, the proteins shown in Supplemental Figure S4 to be involved in the autophagic degradation of protein aggregates. The immunoblot analysis demonstrated that β-escin treatment decreased both SQSMT1/p62 and the ratio of LC3A/B in mesothelial cells and fibroblasts (Supplementary Figure S6B). β-escin-mediated autophagy was further confirmed qualitatively using CYTO-ID staining, which showed that β-escin and Digitoxin induced the expression of autophagocytic vesicles (Supplemental Figure S6C). 

### 2.5. β-escin Inhibits Ovarian Cancer Metastasis In Vivo

**Figure 5:**
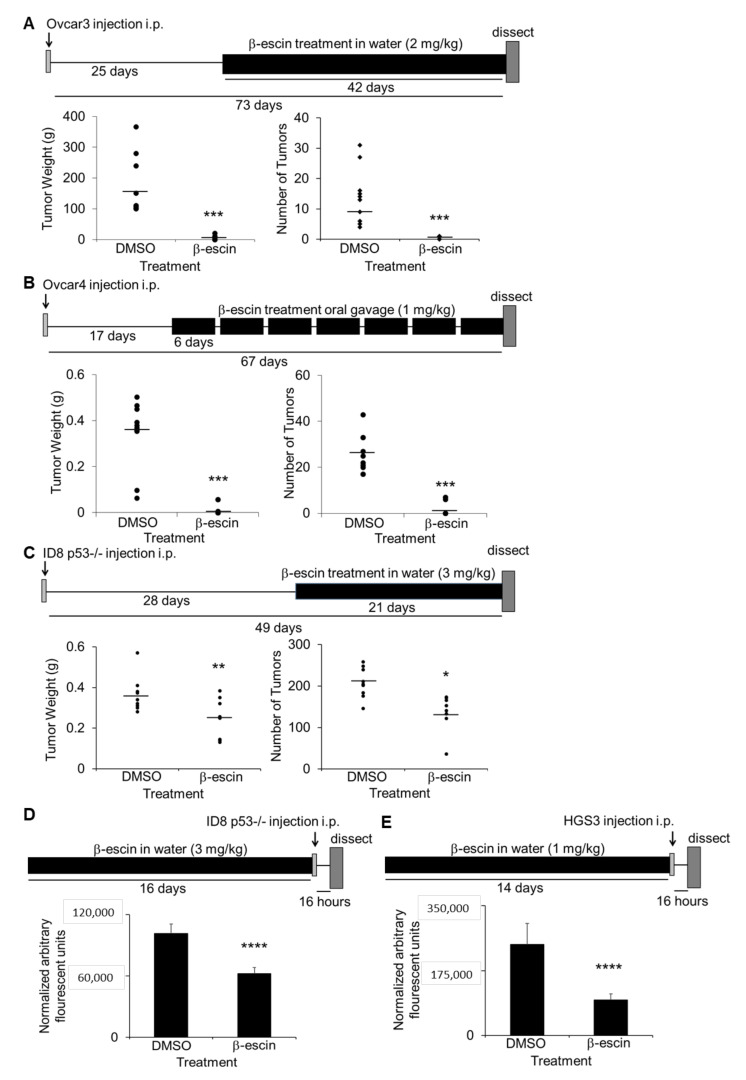
β-escin inhibits ovarian cancer metastasis in vivo. (**A**–**C**) In vivo intervention metastasis assays. The total tumor weight and the number was determined (circles: individual mouse results; line: mean). *, *p* < 0.05; **, *p* < 0.01; ****, *p* < 0.001. (**A**) Twenty-five days after the intraperitoneal injection of Ovcar3 cells (4 million), athymic nude mice were given water ad libitum with β-escin (2 mg/kg/day) or DMSO (50 μL equal volume) in 500 mL total volume, and was sacrificed 42 days later. (**B**) Seventeen days after intraperitoneal injection of Ovcar4 cells (4 million), athymic nude mice were treated six days a week by oral gavage with β-escin (1 mg/kg/day) or DMSO (1 μL equal volume) in PBS (100 μL total) and sacrificed 50 days later. (**C**) Twenty-eight days after intraperitoneal injection of ID8^p53-/-^ (5 million), mouse ovarian cancer cells, C57Bl/6 mice were given water ad libitum with β-escin (3 mg/kg/day) or DMSO (50 μL equal volume) in 500 mL of total volume, and sacrificed 21 days later. (**D,E**) In vivo prevention assay for adhesion/invasion. Fourteen days after the C57BL/6 mice were given water ad libitum with β-escin (1 mg/kg/day) or DMSO (50 μL equal volume) in 500 mL total volume, the mice received an intraperitoneal injection of luciferase-labeled ID8^p53-/-^ cells (5 million) (**D**) or CMFDA-labeled HGS3 cells (5 million) (**E**) of mouse ovarian cancer cells. The mice were sacrificed 16 h post cancer cell injection and the luciferase signal (**D**) or the fluorescence signal (**E**) in the omentum was measured (mean ± SD). ****, *p* < 0.001.

The in vivo efficacy of β-escin as a therapeutic treatment for ovarian cancer metastasis was evaluated. Three intervention treatment and two prevention treatment studies in OvCa metastatic mouse models were performed [25]. First, athymic nude mice were injected intraperitoneally with Ovcar3 cells, and were allowed to establish solid tumors for 25 days prior to treatment with water ad libitum containing β-escin (2 mg/kg/day) for 6 weeks. β-escin treatment resulted in a significantly decreased tumor weight and tumor number (Figure 5A). In a similar approach, 17 days after the intraperitoneal injection of Ovcar4 cells, the mice were treated with β-escin (1 mg/kg/day) for 6 days of each week for 50 days via oral gavage. The mice treated with β-escin using this approach also demonstrated a significantly reduced tumor weight and tumor number (Figure 5B). In the final intervention study, C57Bl/6 immunocompetent mice were injected intraperitoneally with murine ID8^p53-/-^ cells [30], and 28 days after cancer cell injection, the mice were given water ad libitum with β-escin (3 mg/kg/day) for 21 days. The β-escin treatment decreased the tumor weight and tumor number significantly (Figure 5C). In the prevention assays, C57Bl/6 mice were given water ad libitum with β-escin (1 mg/kg/day) for 14 days prior to the intraperitoneal injection of ID8^p53-/-^ GFP cells or CMFDA-labeled HGS3 cells. The β-escin pretreatment also decreased the tumor adhesion/invasion on the mouse omentum significantly (Figure 5D,E).

**Figure 6:**
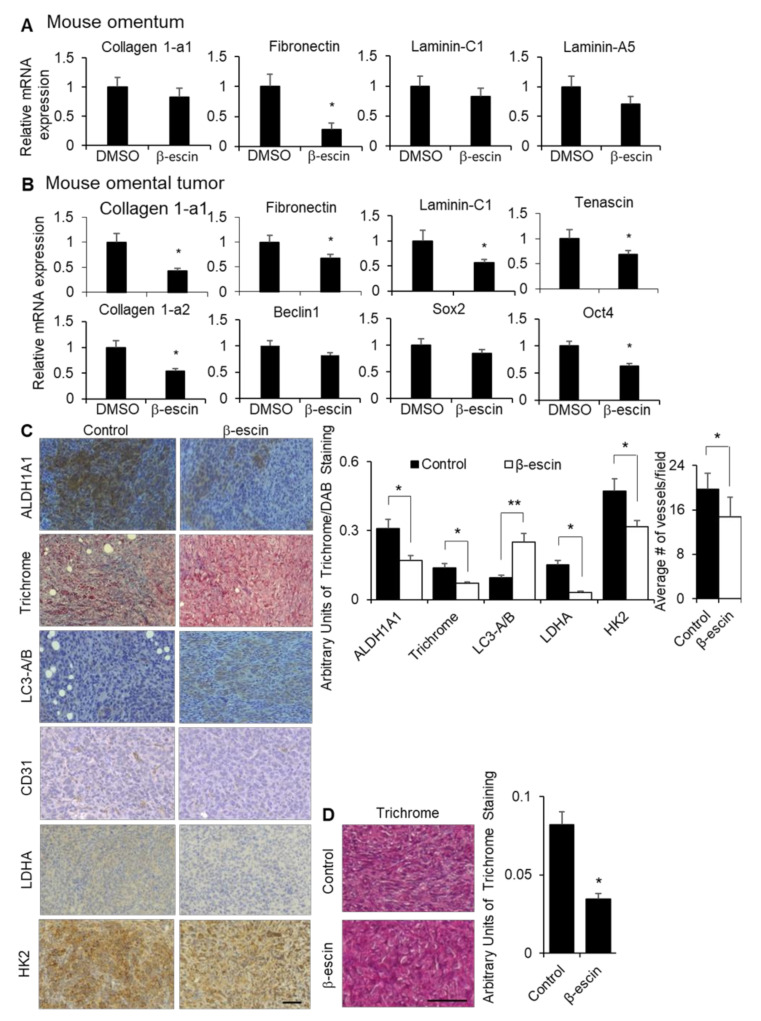
β-escin treatment inhibits extracellular matrices and HIF1α targets in vivo. (**A**) Fibronectin, collagen 1-a1, laminin-C1, and laminin-A5 mRNA expressions were measured by qRT-PCR in mouse omentum after the mice were treated with β-escin (5 mg/kg/day) for 14 days. (**B**,**C**) Tumors were collected from the syngeneic in vivo intervention study. Twenty-eight days after the intraperitoneal injection of ID8 mouse ovarian cancer cells, C57Bl/6 mice were given water ad libitum with β-escin (3 mg/kg/day) or DMSO (50 μL equal volume) in 500 mL of the total volume, and were sacrificed 21 days later when the omental tumor was collected. (**B**) Fibronectin, collagen 1-a1, fibronectin, laminin-C1, tenascin, collagen 1a-2, Beclin1, Sox2, and Oct4 mRNA expressions were measured by qRT-PCR in the mouse omental tumors. (**C**) ALDH1A1, LC3B, LDHA, and HK2 expression levels and the number of vessels (CD31+) were detected by immunohistochemical analyses, while the collagen fibers were stained with trichrome stain (blue) in cross-sections of omental tumors (mean ± SD). *, *p* < 0.05. line, 100 μm. (**D**) Twenty-eight days after the intraperitoneal injection of ID8 mouse ovarian cancer cells, C57Bl/6 mice were given water ad libitum with β-escin (1 mg/kg/day) or DMSO (50 μL equal volume) in 500 mL total volume, and were sacrificed 14 days later when the bowel tumor was collected. The collagen fibers were stained with trichrome stain (blue) in cross-sections of the bowel tumors (mean ± SD). *, *p* < 0.05. **, *p* < 0.01. line, 100 μm.

To confirm the observed in vitro effects of β-escin on the ECM expression, stem cell populations, HIF1α production and stability, and autophagy in vivo, we evaluated the effect of β-escin treatment in mice. The fibronectin expression was significantly decreased in the normal omenta of the mice with β-escin treatment, while the collagen 1-a1, laminin-C1, and laminin-a5 expression were unaffected (Figure 6A). In the tumor transformed area of the mouse omenta from the same mice shown in Figure 5C, the mRNA expression levels of fibronectin, collagen 1-a1, laminin-C1, tenascin, and collagen 1-a2, as well as the stem cell transcription factor Oct4, were significantly decreased. In contrast, the expression levels of Beclin1, a gene involved in autophagy, and Sox2, a stem cell transcription factor, were unchanged following β-escin treatment (Figure 6B). Staining of omental tumors collected from the β-escin treated ID8^p53-/-^ mice, as depicted in Figure 5C, showed an enrichment of LC3A/B, and the concomitant reduction of collagen fiber deposition and the stem marker aldehyde dehydrogenase 1A1 (ALDH1A1). In addition, the HIF1α targets, lactate dehydrogenase (LDHA), hexokinase 2 (HK2), and blood vessels (CD31+) were significantly reduced in these omental tumors after β-escin treatment (Figure 6C). In an additional mouse study, the mice were injected intraperitoneally with ID8^p53-/-^ cells and, after tumor establishment, were treated with β-escin in water given ad libitum for 14 days. Once more, β-escin treatment diminished the collagen fiber deposition in tumors (Figure 6D). 

## 3. Discussion

β-escin is a triterpenoid saponin from the seed of the horse chestnut tree, *Aesculus hippocastanum*, and is an example of a low-cost, over the counter, and non-toxic natural product. Our experiments in OvCa metastasis found multiple lines of evidence that support a therapeutic role for β-escin. OvCa metastasis was efficiently reduced when β-escin oral treatment was given after tumor formation (intervention setting) in xenograft and syngeneic mouse models. Furthermore, β-escin treatment given prior to metastasis (prevention setting) decreased omental metastasis and colonization in syngeneic mouse models. These data complement previous reports of the therapeutic potential of β-escin in other cancers. In the intervention treatment setting, β-escin effectively inhibited bladder tumor growth [11], hepatocellular carcinoma growth [10], and subcutaneous tumor growth of a pancreatic cancer cell line [4] in xenograft mouse models. In the prevention treatment setting, β-escin efficiently prevented the formation of azoxymethane-induced colonic aberrant crypt foci [7], suppressed the formation of tobacco carcinogen 4-(methylnitrosamino)-1-(3-pyridyl)-1-butanone-induced lung adenoma and adenocarcinoa formation [8], and inhibited breast cancer metastasis [14] in rodent models. These studies clearly show that β-escin can prevent cancer development and inhibit cancer growth in vivo in rodents. 

Our high-throughput screening platform identified and validated structurally similar compounds to β-escin that inhibit OvCa cell interaction with the tumor microenvironment. Digitoxin and Ouabain, both cardiac glycosides, were identified and validated to inhibit OvCa adhesion, invasion, migration, proliferation, and metastasis. These cardiac glycosides are plant-derived natural products with known cellular functions, including inhibiting stem cell populations and HIF1α stability, while enhancing autophagy [31,32,33]. β-escin selectively targeted the stem cell population of glioblastoma multiforme [34], and induced autophagy in osteosarcoma cells [15]. In contrast, in the present study, β-escin did not affect HIF1α production in ovarian cancer cells, but did regulate HIF1α stability. Two reports in triple-negative breast cancer found that β-escin did not regulate hypoxia-induced HIF1α [14]. However, in non-small cell lung cancer, β-escin inhibited the stabilization of HIF1α by hypoxia [35]. In addition, in our study, the treatment of mice with β-escin led to a decrease in the HIF1α targeted genes LDHA, CD31, and HK2 in vivo. Because HIF1α is a notable transcription factor driving both autophagy and the associated CSC differentiation [36,37], it is plausible that the inhibition of the autophagy and CSC markers by β-escin observed here are linked to its inhibition of HIF1α. We also observed that β-escin repressed HIF1α, as well as a number of key ECM proteins in fibroblasts and mesothelial cells, which was linked with the decreased ability of OvCa cells to adhere to and invade the TME. This is consistent with the reported necessity of HIFs for promoting ECM production [38] and cancer cell adhesion to extracellular components such as fibronectin [39], as well as the putative role of HIF activity in driving the progression of multiple types of cancer through a number of other mechanisms [31]. Taken together, these data suggest that β-escin may prevent metastasis in OvCa by preventing autophagy-dependent CSC differentiation and stromal ECM production driven by HIF1α.

Treatment with β-escin also imitates a function of cardiac glycosides by inhibiting ATP-dependent sodium−potassium exchange across cell *membranes* [32]. In this study, we demonstrate the ability of β-escin to mimic cardiac glycosides by inhibiting sodium-potassium exchange across the plasma membrane. Although this effect of β-escin treatment is not directly reported in previous literature, β-escin can enhance nitric oxide in cells [33] that are known to decrease the molecular activity of Na^+^,K^+^-ATPase [34]. Extensive studies have shown that cardiac glycosides directly bind to Na^+^,K^+^ ATPase, changing the enzymes’ ability to pump ions and assemble multiple protein complexes [35,40]. Evidently, cardiac glycosides prevent the interaction of Src with the enzyme, leading to the activation of multiple protein kinase cascades, including PI3K, Phospholipase C, and MAPK signaling, and ultimately the apoptosis of cancer cells. In future studies, we will focus on investigating whether β-escin parallels cardiac glycoside-intracellular signaling events. β-escin did not inhibit ATP-dependent sodium−potassium exchange across cell membranes in the mouse OvCa cell line. Similarly, the inhibitory activity of cardiac glycosides on Na^+^,K^+^-ATPase is 1000 times more sensitive in human cells than mouse cells because of the species differences in the α1-isoform of the enzyme [41]. Therefore, the inhibitory activity of β-escin on Na^+^,K^+^-ATPase is specific to human OvCa cells, and further analysis is needed to explore the NKA-independent effects of β-escin in mice. In addition, the ability of β-escin to inhibit OvCa cell adhesion in vitro in the present study may be explained, in part, through its inhibition of Na^+^,K^+^-ATPase, which acts as a cell adhesion molecule in adherens junctions. This inhibitory effect is associated with Na^+^,K^+^-ATPase’s ability to promote cancer cell adhesion and motility [42]. However, further investigation is needed to link Na^+^,K^+^-ATPase activity with cancer cell adhesion and/or invasion, as well as to determine the effect of β-escin on Na^+^,K^+^-ATPase-dependent processes. 

While in vitro and in vivo data support the possible anti-tumor effects of β-escin and the cardiac glycosides analyzed in the present study, there are conflicting data regarding their therapeutic efficacy. In a recent retrospective meta-analysis, it was shown that cardiac glycoside use was associated with a slight increase in the incidence of breast cancer but a lower risk of developing advanced prostate cancer [43]. Additionally, an earlier retrospective report showed that Digoxin was not associated with a significant effect on outcomes in patients with epithelial ovarian cancer [44]. Limited data exist on the effects of β-escin in cancer; however, a prospective clinical trial reported its therapeutic potential in improving progression-free survival in patients with advanced thyroid cancer [45]. Without prospective phase II clinical trials that compare β-escin treatment to standard of care, it is difficult to determine what impact this agent may have on OvCa progression in patients.

## 4. Materials and Methods

### 4.1. Reagents

Antibodies for GAPDH (#2118), HIF1α (#14179), LC3A/B (#12741), SQSTM1/p62 (#5114), Cyclin-D1 (#2978; 92G2), E-Cadherin (#3195), and horse radish peroxidase-linked anti-mouse (#7076) and anti-rabbit (#7074) IgG were acquired from Cell Signaling Technology (Danvers, MA, USA). Antibodies for fibronectin were purchased from Santa Cruz Biotechology Inc. (#sc-8422; clone EP5; Dallas, TX, USA), and β-actin from Sigma Aldrich (#a5441; St. Louis, MO, USA). β-escin was purchased from Sigma Aldrich (SA; #E1378), MP Biomedicals (MPB; 02157941), and Santa Cruz (SC; sc-221596). Horse chestnut extract was purchased from Solaray (Salt Lake City, UT, USA). A structural similarity search using the 2D Tanimoto coefficient was used to pull out 160 compounds that had ≥65% structural similarity (Tanimoto coefficient ≥ 0.65) to β-escin in order to cover a diverse compound set for follow-up studies. The 160 structurally similar compounds of β-escin were cherry-picked from the screening libraries housed at the NCATS. 

### 4.2. Cell Lines

The high-grade serous OvCa cell lines HeyA8, SNU-119, Kuramochi, Ovcar4, and Ovcar5 were provided by the University of Texas MD Anderson Cancer Center (Houston, TX, USA), Korean Cell Line Bank (#00119; Copenhagen, Denmark), JCRB Cell Bank (#JCRB0098; Osaka, Japan), DCTD Tumor/Cell Line Repository (#0507673; Federickm, MD, USA), and UCSF (San Francisco, CA, USA), respectively. The COV318, TYK-nu, and OVKATE cells were obtained from Gottfried Koneczny, originally at UCLA. The ID8^p53-/-^ were provided by Iain McNeish at the Imperial College of London [30]. The CaOV3 and SKOV3ip1 cells were purchased from the ATCC (#HTB-75; #HTB-77). All of the cell lines were passaged three to eight times after thawing before use in the described experiments. The cell lines were banked in liquid nitrogen and were confirmed to be mycoplasma negative using the STAT-Myco Kit and were validated using short tandem repeat DNA fingerprinting with the AmpF‘STR Identifier Kit and compared with known fingerprints by IDEXX BioAnalytics Laboratories. Five or six high-grade serous OvCa cell lines were utilized for the confirmatory assays to identify the compounds that are active in all of these cell lines. Three high-grade serous OvCa cell lines (Tyk-nu, Ovcar5, and Kuramochi) were chosen to perform the secondary functional assays because of their differential activity in these functional assays. Ultimately, we wanted to confirm if the compounds tested were active in different OvCa cell lines, regardless of whether they were more or less adhesive, invasive, or proliferative. 

### 4.3. Primary Human Mesothelial Cell and Fibroblast—Isolation and Culture

Specimens of fresh human omentum were obtained from patients undergoing surgery for benign conditions, who gave written informed consent before surgery. The protocol was approved by the University of Chicago Institutional Review Board (IRB 13372B). Our study involving the collection of omentum or omental tumors from patients follows the rules of the Declaration of Helsinki. Primary human mesothelial cells and fibroblasts were isolated from normal human omentum; purity was verified by vimentin, calretinin, and cytokeratin IHC [21,25,46].

Primary cells were used for experiments at passages 1–2 to minimize any divergence from the original characteristics and morphology [47]. 

### 4.4. Primary 3D HTS (3D-qHTS) Assay

For the 1536-well format, 40 primary human omental fibroblasts and 400 mesothelial cells were seeded with 0.02 μg fibronectin and 0.02 μg collagen type I in 4 μL of growth media (2.3 mm^2^) [6]. After a 48-h incubation at 37 °C, 1200 SKOV3ip1-GFP were seeded in 3 μL of serum-free media (growth media minus FBS) on top of the primary human omental cells. The compounds were screened in four doses (0.36–46 μmol/L), and the plates contained the positive control (Tomatine) in eight doses (0.035–75 μmol/L) and dimethyl sulfoxide (DMSO; equal volume controls). The compounds or controls were added to each well immediately after the addition of cancer cells. The plates were incubated at room temperature for 2 h to reduce well-to-well variability and edge effects [48], and then at 37 °C for 16 h to allow the cells to adhere and invade the primary human omental cells. Following incubation, each well was washed with PBS (5 μL) and fixed with 4% paraformaldehyde (PFA; 5 μL). After 15 min, the PFA was removed, PBS (5 μL) was added, and the number of GFP-labeled cells were analyzed using a fluorescent cytometer (TTP LabTech Acumen eX3; Tokyo, Japan).

### 4.5. Confirmatory and Counter Assay

The 3D-qHTS assay was plated in a 384-well format. Primary human omental fibroblasts (400) and mesothelial cells (4000) were seeded with collagen type I in 40 μL of growth media (0.06 cm^2^). After 48 h of incubation at 37 °C/5% CO_2_/95% relative humidity, SKOV3ip1-GFP, HeyA8-GFP, Kuramochi-GFP, COV318-GFP, SNU119-GFP, Tyk-nu-GFP, or Ovcar4-GFP OvCa cells (12,000) were seeded in 40 μL of serum-free media (growth media minus FBS) on top of the primary human omental cells (fibroblasts and mesothelial cells; final volume of 80 μL total). The library compounds dissolved in DMSO at a 46 μmol/L final concentration, positive control (Tomatine) at 10 μmol/L final concentration, or DMSO (0.5% final concentration) were added to each well immediately after the addition of the cancer cells. The plates were incubated at room temperature for 2 h before being placed in an incubator at 37 °C. After a 16-h of incubation, each well was washed with PBS (40 μL), followed by fixation with 4% paraformaldehyde. The number of fluorescent cells in the assay was analyzed using a fluorescent cytometer (TTP Labtech Acumen eX3). 

For the confirmatory assay, quality-checked compounds were cherry-picked and the dose−response of the compounds was tested using 11 concentrations with 1:3 dilutions (0.8–46 μmol/L final concentration). The compounds dissolved in DMSO or DMSO (equal volume control) were added to each well immediately after the addition of cancer cells. The plates were incubated, treated, and fixed, as described above. The number of fluorescent cells were analyzed using the fluorescent cytometer.

For the counter assay, the OvCa cells (4000 SKOV3ip1-GFP or 2000 HeyA8-GFP) were seeded in 40 μL of growth media and incubated for 24 h. Compounds dissolved in DMSO at 12 concentrations (10–100 μmol/L) or DMSO (equal volume control) were added. The plates were incubated at room temperature for 2 h before being placed in an incubator at 37 °C. After a 16-h incubation at 37 °C, the CellTiter-Glo cell viability assay (Promega) was performed and analyzed using a luminescent plate reader (BioTek Synergy NEO2; Winoski, VT, USA).

The compounds’ area under the curve (AUC) for the activity outcomes from the confirmatory and counter screens were calculated on the basis of the data analysis and dose−response curve fittings. The compounds were clustered hierarchically using TIBCO Spotfire 6.0.0 (Spotfire Inc., Somerville, MA, USA).

### 4.6. Secondary Biological In Vitro Assays

The secondary biological assays were miniaturized for high-throughput analysis. The 3D culture was assembled on black-walled 384-well plates for proliferation assays using the iPipette from Apricot Designs. For the invasion assays, the 3D culture was assembled on precoated (7 μg of collagen type I) 96-well transwell inserts (BD Biosciences; Franklin Lakes, NJ, USA; [6]). The compounds were purchased as described above. The β-escin sources were tested at 0.1, 0.5, 1.0, 5, and 10 μmol/L; compounds dissolved in DMSO were tested at 1, 2, 5, and 10 μmol/L concentrations; and DMSO (equal volumes) was the control. 

*Invasion:* A total of 8000 Tyk-nu-GFP, Ovcar5-GFP, or Kuramochi-GFP cells were seeded in 40 μL of serum-free media in the upper chamber of a 96-well transwell plate (0.134 cm^2^, *n* = 5–10) precoated with the 3D culture [6]. The compounds were added to the cancer cells in the upper chamber, growth media (100 μL) were placed in the bottom chamber, and the plates were incubated at 37 °C for 24 to 48 h. All of the cells were removed from the top chamber and the invaded OvCa cells were quantified using the SpectraMax i3 MiniMax 300 imaging cytometer.

*Proliferation/number of cells over time analysis*: A total of 2000 Tyk-nu-GFP, 1000 Ovcar5-GFP, or 4000 Kuramochi-GFP cells were seeded in 40 μL of serum-free media on top of the 3D culture (0.33 cm^2^, *n* = 5–15). The compounds were added after 30 min, plates were incubated for 96 h at 37 °C, and the total number of cells was counted using the SpetraMax i3 MiniMax 300 imaging cytometer.

### 4.7. Animal Experiments

Female C57BL/6NCrl (C57BL/6; #027) mice and female HSD: Athymic Nude-Foxn1^nu^ (athymic nude) mice aged 5 to 6 weeks and approximately 20 g were purchased from Charles River Laboratories. All of the procedures involving animal care were approved by the Institutional Animal Care and Use Committee at the University of Chicago (Chicago, IL, ACUP 71951).

*Secondary biological screen*: in vivo adhesion/invasion assay [6]. C57Bl/6 mice were randomized into groups (*n* = 5) and injected intraperitoneally with 5 × 10^6^ ID8^p53−/−^ GFP cells mixed with compounds (5 μmol/L) or DMSO (1 μL equal volume) in cold-phosphate buffered saline (500 μL total). Sixteen hours (the same time span as the primary screen) after cancer cell injection, the mice were sacrificed. The omentum was removed, omental lysates were prepared, and the total fluorescence was quantified using the Spectramax (Molecular Devices) imaging cytometer, as described previously [6].

*Secondary biological screen*: in vivo prevention metastasis assay. The C57Bl/6 mice were randomized into groups (*n* = 5) and injected intraperitoneally with 5 × 10^6^ ID8^p53-/-^ cells mixed with compounds (5 μmol/L) or DMSO (1 μL equal volume) on day 1 (five mice/group). The mice were additionally treated with compounds (1 mg/kg/day) or DMSO (1 μL equal volume) in PBS (100 μL total) on days 2 and 4. Forty-five days after injection, the mice were sacrificed. The tumor colonies located in the liver, bowel, and reproductive tract mesentary and omentum, as well as on the diagphram and abdominal wall/peritoneum, were counted, collected, and weighed [6].

*Ovcar3*: in vivo intervention metastasis assay. Athymic nude mice were randomized into groups (*n* = 5) and injected intraperitoneally with 4 × 10^6^ Ovcar3 cells. Twenty-five days later, the mice (5 mice/group) were given water ab libitum with β-escin (2 mg/kg/day) or DMSO (50 μL equal volume) in 500 mL of total volume. The mice were sacrificed 42 days after treatment or 73 days after cancer cell injection. The tumor colonies located in the liver and bowel mesentery, and the omentum, as well as on the diagphram and abdominal wall/peritoneum, were counted, collected, and weighed. 

*Ovcar4*: in vivo intervention metastasis assay. Athymic nude mice were randomized into groups (*n* = 5) and injected intraperitoneally with 4 × 10^6^ Ovcar4 cells. Seventeen days later, the mice were treated by oral gavage with β-escin (1 mg/kg/day) or DMSO (1 μL equal volume) in PBS (100 μL total) for 6 days a week. The mice were sacrificed 45 days after treatment or 67 days after cancer cell injection. The tumor colonies located in the liver and bowel mesentery, and omentum, as well as on the diagphram and abdominal wall/peritoneum, were counted, collected, and weighed. 

*ID8*: in vivo intervention metastasis assay. C57Bl/6 mice were randomized into groups (*n* = 5) and injected intraperitoneally with 5 × 10^6^ ID8^p53-/-^ cells. Twenty-eight days later, the mice were given water ad libitum with β-escin (3 mg/kg/day) or DMSO (50 μL equal volume) in 500 mL total volume, and were sacrificed 21 days later. The tumor colonies located in the liver, bowel, and reproductive tract mesentary and omentum, as well as on the diagphram and abdominal wall/peritoneum, were counted, collected, and weighed.

*ID8*: in vivo prevention treatment followed by adhesion/invasion assay. C57Bl/6 mice were randomized into groups (*n* = 5) and given water ad libitum with β-escin (1 mg/kg/day) or DMSO (50 μL equal volume) in 500 mL total volume. Fourteen days later, the mice were injected intraperitoneally with 5 × 10^6^ ID8^p53-/-^ GFP cells. Sixteen hours after cancer cell injection, the mice were sacrificed. The omentum was removed, omental lysates were prepared, and the total fluorescence was quantified using the Spectramax (Molecular Devices) imaging cytometer, as described previously [6].

*HGS3*: in vivo prevention treatment followed by adhesion/invasion assay. C57Bl/6 mice were randomized into groups (*n* = 5) and given water ad libitum with β-escin (3 mg/kg/day) or DMSO (50 μL equal volume) in 500 mL of total volume. Fourteen days later, the mice were injected intraperitoneally with 5 × 10^6^ CMFDA-labeled *HGS3* cells [49]. Sixteen hours after cancer cell injection, the mice were sacrificed. The omentum was removed, omental lysates were prepared, and the total fluorescence was quantified using the Spectramax (Molecular Devices) imaging cytometer, as described previously [6].

### 4.8. Electrophysiology

SKOV3ip1, Tyk-nu, and ID8^p53-/-^ cells were plated on glass coverslips and incubated overnight at 37  °C. For each coverslip, one healthy cell was selected based on its morphology. This healthy cell was patch-clamped with a glass micro pipet. The pipet measured 5–7 MΩ when filled with 20 mM tetramethylammonium hydroxide (TMA-OH), 90 mM NaOH, 20 mM tetraethylammonium chloride (TEA-CL), aspartic acid, 2 mM MgCl_2_, 5 mM EGTA, 5 mM Tris-ATP, 2.5 mM Tris-creatine phosphate, 5 mM glucose, and 10 mM HEPES at pH 7.4. The bath solution used to perfusate the cells was composed of 40 mM NaCl, 5.4 mM KCl, 0.5 mM MgCl_2_, 0.33 mM NaH_2_PO_4_, 5.5 mM glucose, 5 mM HEPES, 2 mM BaCl_2_, 1 mM CsCl, 0.2 mM CdCl_2_, 2 mM NiCl_2_, and 1 μM nifedipine at pH 7.4. This combination of pipette and bath solution allowed us to isolate the Na^+^,K^+^-ATPase current [50]. All of the recordings were performed in the whole-cell configuration. The cells were gravity-fed (VC-8, Warner Instruments) with a bath solution until a stable reading for the current was recorded. Once the current was stable, the cell was perfused with 2 µM (Tyk-nu) or 10 μM (SKOV3ip1) β-escin. The recordings were made using a 2 s ramp protocol from 50 mV to −160 mV, applied from a holding potential of −60 mV. All of the recordings were performed at room temperature using an electrophysiology rig built around a Nikon Eclipse TE300 inverted microscope (Nikon; Mellville, NY, USA), PATCHSTAR micropositioner (Scientifica, East Sussex, UK), low noise amplifier Axopatch 200B, and D/A converter Digidata 1550B, and the data were acquired using pClamp-10 software and analyzed using Clampfit software (all from Molecular Devices, Sunnyvale, CA). All the plots were generated and compared for statistical significance using normalized current response in Origin Pro2018 (OriginLab Corp., Northampton, MA, USA) for each cell.

### 4.9. Aldehyde Dehydrogenase Activity Assay 

OvCa cells (Tyk-nu 4000, Ovcar5 2000, and Kuramochi 8000) were grown in 5% oxygen as spheroids in an ultra-low attachment round bottom 96-well plate for 96 h. The cells were treated with β-escin (10 μM), Digitoxin (0.002 μM), or DMSO (equal volume) for 48 h at 37 °C/5% O_2_/5% CO_2_. All of the cells were washed in PBS and dissociated with 0.25 trypsin-EDTA (Gibco, Carlsbad, CA, USA). The ALDEFLUOR™ Assay kit (STEMCELL Technologies; Cambridge, MA, USA) was used following the manufacturer’s instructions. Negative control using diethylaminobenzaldehyde (DEAB; a specific inhibitor of ALDH1 activity) was prepared for each sample to correct the fluorescence background. Data analysis was performed using FlowJo Software by establishing the sorting gates relative to the background fluorescence of the DEAB-treated samples. The ALDH1 activity was normalized relative to the control without treatment/solvent. Three independent experiments were conducted in duplicate.

### 4.10. Immunoblots

The cell lysates were prepared in a RIPA buffer (100 mM Tris/HCl (pH 7.4), 150 mM NaCl, 1 mM EDTA, 0.1% (*w*/*v*) sodium dodecyl sulfate, 0.5% sodium deoxycholate, and 1% Triton X-100, and supplemented with phosphatase/protease inhibitors immediately prior to use). Equal amounts of protein were added to each well of the SDS-PAGE gel and resolved. The proteins were transferred to a nitrocellulose membrane. The membranes were blocked in 5% milk tris-buffered saline with 0.1% tween. The membranes were incubated in primary antibodies at a 1:1000 dilution in 5% BSA Tris-buffered saline overnight at 4 °C. The next day, the blot was incubated with horseradish peroxidase-conjugated IgG secondary antibody at room temperature for 1 h at a 1:5000 dilution and visualized using chemiluminescence reagents and the G:BOX Chemi XT4 imager (Syngene; Cambridge, UK). 

### 4.11. Proteomics

The OvCa (Tyk-nu) cells were seeded on the bottom of a 0.4 μm pore-size PET filter, and primary human mesothelial cells were seeded on the top of this filter to allow for the exchange of secreted factors between the two cell types. The primary human mesothelial cells were collected from the top chamber and proteomics analysis was performed using a Q-Exactive HF mass spectrometer with EASY-nLC 1000 HPLC system (ThermoFisher Scientific; Waltham, MA, USA), as described previously [20,29]. Data were acquired using Xcalibur (ThermoFisher) and were analyzed using MaxQuant (Max Planck Institute; Munich, Germany) [51]. Statistical analyses were carried out using Perseus, with statistically significant proteins identified by Benjamini−Hochberg cutoff, with a false discovery rate (FDR) of 0.05 and S_0_ of 0.1. The pathway enrichment analysis was assessed using the Molecular Signature Database (UCSD Broad Institute, San Diego, CA, USA). 

### 4.12. Quantitative Real-Time Polymerase Chain Reactions

After the treatment of OvCa cells with 1–10 μM β-escin for 36 h, mouse omentum or omental tumors were collected from the described animal experiments. The TRIzol reagent was used to isolate the RNA according to the manufacturer’s instructions (Invitrogen). cDNA was synthesized using the Applied Biosystems cDNA archive kit. After reverse transcription, real-time PCR was performed using a Prism7500 TaqMan PCR detector (Applied Biosystems; Waltham, MA, USA) with predesigned and validated TaqMan probes for Sox2, Oct4, KLF4, HIF1α, tenascin C, collagen 1-a1, fibronectin, vitronectin, laminin-C1, laminin-a5, collagen 1-a1, and Beclin1 in conjunction with GAPDH for normalization (Applied Biosystems) [15]. The reactions were run in triplicate. Relative levels of mRNA expression were calculated using the 2^–ΔΔCt^ method [52]. Differences between treatments were evaluated using unpaired two-tailed Student’s *t*-test.

### 4.13. Immunohistochemistry/Trichrome Staining

For the immunohistochemical experiments, mouse bowel tumors were fixed in 10% formalin for 24 h and were paraffin-embedded. The paraffin blocks were cut onto Superfrost Plus charged slides (Thermo Fisher Scientific, Waltham, MA, USA), deparaffinized in xylene, and hydrated with alcohol. The peroxidase activity was quenched with a 3% H_2_O_2_/methanol blocking solution for 30 min. The slides were boiled in 0.01 M sodium citrate pH 5.0 for 20 min to retrieve the antigens. Cells cultured on the chamber slides were fixed in acetone/methanol (1:1). For immunohistochemistry, all of the slides were blocked in avidin and biotin blocking solutions (Vector Laboratories, Burlingame, CA, USA). The slides were incubated with the primary antibodies overnight at 4 °C. After 3 washes in tris-buffered saline, the slides were incubated with a biotinylated secondary antibody (1:200). The slides were washed again with tris-buffered saline and then incubated with peroxidase-linked avidin using the Vectastain ABC kit (Vector Laboratories, Burlingame, CA, USA) for 30 min. The slides were rinsed in tris-buffered saline and stained with 3-3′-diaminobenzidine chromogen, then counterstained with hematoxylin. Appropriate negative controls for the immunostaining were prepared by omitting the primary antibody. Trichrome staining to detect collagen fibers was conducted as previously published [21].

### 4.14. ODD-Luciferase Reporter Assays

The ODD-luciferase reporter construct consisting of the firefly luciferase gene fused to the hydroxylation-dependent degradation region of HIF1a was purchased from Addgene (18965). The ODD-luciferase reporter plasmid was transiently transfected into Tyk-nu, Ovcar5, or Kuramochi OvCa cells. Thirty-six hours after transfection, the cells received treatment (no treatment, omental conditioned-media, or 1% oxygen). The omental conditioned media was used to partially reproduce the microenvironment (secretome) that OvCa cells are exposed to during ometnal metastasis. Eight hours after treatment, the cells were lysed and a luciferase assay (Promega) was performed according to the manufacturer instructions. The luciferase signal was normalized to the total protein. The total protein concentration was obtained using a BCA protein assay (Thermo Fisher Scientific).

### 4.15. Statistical Analysis

Confirmatory (*n* = 8), adhesion (*n* = 8), invasion (*n* = 8), and growth (*n* = 8) assays were conducted in at least three independent experiments. The mean and SD or SEM are reported. All of the statistical analyses were performed using GraphPad Prism (GraphPad). For the trials comparing two groups, data were analyzed using a two-tailed Mann–Whitney U test to account for the non-normal distribution of the data. For experiments with more than two groups, one-way ANOVA followed by Dunnett multiple comparisons test (DMSO vs. each of the other groups) was used. Differences were considered significant if *p* < 0.05.

## 5. Conclusions

OvCa metastasizes to mesothelial cell-lined organs in the peritoneal cavity [21]. OvCa cells induce mesothelial cell EMT, promoting ECM secretion (i.e., fibronectin) and the subsequent progression of tumor growth and metastasis [25,40]. Our findings show that in vitro and in vivo treatment with β-escin represses the EMT pathway and ECM production in the mesothelial cells and fibroblasts. This is the first report on the effect of β-escin on the stromal component of the tumor microenvironment, further explaining its anti-tumor effect.

Our studies, as well as previous literature [27,39], support the exploration of β-escin and the cardiac glycosides, Ouabain and Digitoxin, as cancer therapeutics. Cardiac glycosides can often be cytotoxic, with narrow therapeutic windows [40]. Conversely, β-escin and horse chestnut seed extract have been tested in clinical trials and are well tolerated in patients. Currently, β-escin is used as an anti-inflammatory, anti-edematous, antioxidant, antiseptic, analgesic, antipyretic, and anti-hemorrhoidal agent [41,42]. The results of our study indicate that β-escin could be a new, cost-effective, and readily available preventative and therapeutic agent for OvCa patients. The additive or synergistic effect of β-escin with platinum-based and other first-line therapies for OvCa patients still need to be examined.

## Supplementary Materials

The following are available online at https://www.mdpi.com/article/10.3390/cancers13163931/s1. Supplementary Materials and Methods: Supplementary Tables S1 and S2: Enrichment analysis and proteomics data of primary human mesothelial cells co-cultured with Tyk-nu ovarian cancer cells. Supplementary Table S3: Proteomics data of primary human mesothelial cells treated with DMSO (control) or β-escin. Supplementary Figure S1: Activity of different sources of β-escin on ovarian cancer cell adhesion. Supplementary Figure S2: Quantitative high throughput screening with a 3D organotypic model to identify structurally similar compounds to β-escin that inhibit OvCa adhesion and invasion. Supplementary Figure S3: Activity of β-escin and cardiac glycosides on ovarian cancer cells. Supplementary Figure S4: β-escin regulates SQSTM1/p62 and LC3A/B production in ovarian cancer cells. Supplementary Figure S5: Quality control for proteomics. Supplementary Figure S6: β-escin activates autophagy in mesothelial cells. Supplemental Figure S7: Unprocessed immunoblots, densitometry reading, and ratio from Figure 3C. Supplemental Figure S8: Unprocessed immunoblots, densitometry reading, and ratio from Figure 4F. Supplemental Figure S9: Unprocessed immunoblots, densitometry reading, and ratio from Figure 4G. Supplemental Figure S10: Unprocessed immunoblots, densitometry reading, and ratio from Supplemental Figure S4A. Supplemental Figure S11: Unprocessed immunoblots, densitometry reading, and ratio from Supplemental Figure S6A. Supplemental Figure S12: Unprocessed immunoblots, densitometry reading, and ratio from Supplemental Figure S6B. Supplementary Video S1: Ovcar5 migration control/DMSO treatment. Supplementary Video S2: Kuramochi migration control/DMSO treatment. Supplementary Video S3: Ovcar5 migration β-escin treatment. Supplementary Video S4: Kuramochi migration β-escin treatment. Supplementary Video S5: Ovcar5 migration Digitoxin treatment. Supplementary Video S6: Kuramochi migration Digitoxin treatment.
