# Supplementary material for: The Natural Product β-Escin Targets Cancer and Stromal Cells of the Tumor Microenvironment to Inhibit Ovarian Cancer Metastasis
Source: Cancers (Basel). 2021 Aug 4;13(16):3931. doi: 10.3390/cancers13163931 (PMC8394501; doi:10.3390/cancers13163931)
Supplement: Supplementary file 1 [file cancers-13-03931-s001.zip › HAK_supplemental_betaescin_manuscript_07152021.pdf]

Supplemental Materials and Methods

The Natural Product β-Escin Targets Cancer and Stromal Cells of the Tumor Microenvironment to Inhibit Ovarian Cancer Metastasis

Hilary A. Kenny [1], Peter C. Hart [1], Kasjusz Kordylewicz [1], Madhu Lal [2], Min Shen [2], Betul Kara [1], Yen-Ju Chen [1], Niklas Grassl [3], Yousef Alharbi [4], Bikash R. Pattnaik [5], Karen M. Watters [1], Manish S. Patankar [4], Marc Ferrer [2] and Ernst Lengyel [1]

[1] Department of Obstetrics and Gynecology, Section of Gynecologic Oncology, University of Chicago, Chicago, IL 60637, USA
[2] Division of Preclinical Innovation, National Center for Advancing Translational Sciences (NCATS), NIH, Rockville, MD 20852, USA
[3] Department of Proteomics and Signal Transduction, Max Planck Institute of Biochemistry, 82152 Martinsried, Germany
[4] Department of Obstetrics and Gynecology, University of Wisconsin-Madison, Madison, WI 53792, USA
[5] Department of Pediatrics and Ophthalmology and Visual Sciences, University of Wisconsin-Madison, Madison, WI 53706, USA

Materials and methods

Secondary biological *in vitro* adhesion assays

The secondary biological adhesion assays were miniaturized for high-throughput analysis. The 3D culture was assembled on black-walled 384-well plates for adhesion assay using the iPipette from Apricot Designs. The compounds were repurchased as described above. Compounds dissolved in DMSO were tested at 1, 2, 5, and 10 μmol/L concentrations, and DMSO (equal volumes) was the control.

For adhesion assays, a total of 8,000 fluorescently labeled Tyk-nu-GFP, Ovcar5-GFP, or Kuramochi-GFP ovarian cancer cells were mixed with the compounds and seeded in 40 μL of serum-free media on top of the 3D culture (0.33 cm2, n = 5–15). After 1-hour incubation at 37°C, the wells were washed with PBS, fixed with PFA, and cell number computed using a Spectramax i3 MiniMax 300 imaging cytometer (Molecular Devices).

Colony formation assays

Cells were seeded onto 6-well plates and treated with β-escin, Ouabain or Digitoxin for 7 days before fixation, crystal violet staining and imaging (G-box, Syngene). For soft agar colony formation, the cells mixed with 0.4% agar/medium were seeded onto 0.8% agar/medium support in 6-well plates and treated with the liposomes (100 nmol/L paclitaxel) for 40 days with the media changed every week. Cells were fixed, stained with crystal violet, and imaged with the G-Box bioimaging system.

CYTO-ID Autophagy Detection Assay

Cells were seeded onto a glass bottom 35mm dish and incubated for 24 hours. After DMSO, β-escin or Digitoxin treatment for 8 hours at 37°C, the cells were stained with CYTO-ID green reagent and Hoechst 33342 nuclear stain according to manufacturer instructions.

Animal experiments

Female C57BL/6NCrl (C57BL/6; #027) mice at age 5 to 6 weeks and approximately 20 g were purchased from Charles River Laboratories. All procedures involving animal care were approved by the Institutional Animal Care and Use Committee at the University of Chicago (Chicago, IL).

ID8 - In vivo early treatment metastasis assay

C57Bl/6 mice were randomized into groups (n = 5) and received β-escin treatment by oral gavage (5mg/kg). On the third day of treatment, 5 x 10^6 ID8$^{p53-/-}$luciferase/GFP cells were injected intraperitoneally. The mice continued to receive β-escin treatment by oral gavage (5mg/kg) for 6

days a week for 3 additional weeks, followed by no treatment until sacrifice 67 days later. The tumor colonies were counted, collected, and weighed.

Migration wound healing assay

In a 96-well tissue plate, OvCar5, TykNu, and Kuramochi cells were seeded at densities 30,000, 30,000 and 100,000 cells per well (100 μL/well), respectively, with appropriate corresponding cell culture media. The cells were incubated at 37°C until they reached 100% confluence (24-48 hours). Media was then removed and each well was washed twice with sterile phosphate-buffered saline (PBS). The cells were treated with 0.001-60 μmol/L concentrations of β-escin, Digitoxin or Ouabain. DMSO (equal volumes) was the control. The cells were incubated for 1 hour at 37°C, the media was carefully removed and each well was washed once with sterile PBS. After washing, wounds were made using the 96-pin IncuCyte WoundMaker from Essen BioScience (#4563) following the provided protocol. The quality of the wounds were checked under a light microscope and after a successful quality control, the wells were gently washed twice with sterile PBS and replenished with 100 μL of fresh cell culture media mixed with treatment or DMSO. The cell plate was then placed into the IncuCyte S2 Live-Cell Analysis System from Sartorius and images were taken at consistent time intervals with a 4X objective until the wound closed. The distance of the wound was measured over time using ImageJ.

Supplementary Figure Legends

Supplementary Table S1–S2. Enrichment analysis and proteomics data of primary human mesothelial cells co-cultured with Tyk-nu ovarian cancer cells.

Supplementary Table S3. Proteomics data of primary human mesothelial cells treated with DMSO (control) or β-escin.

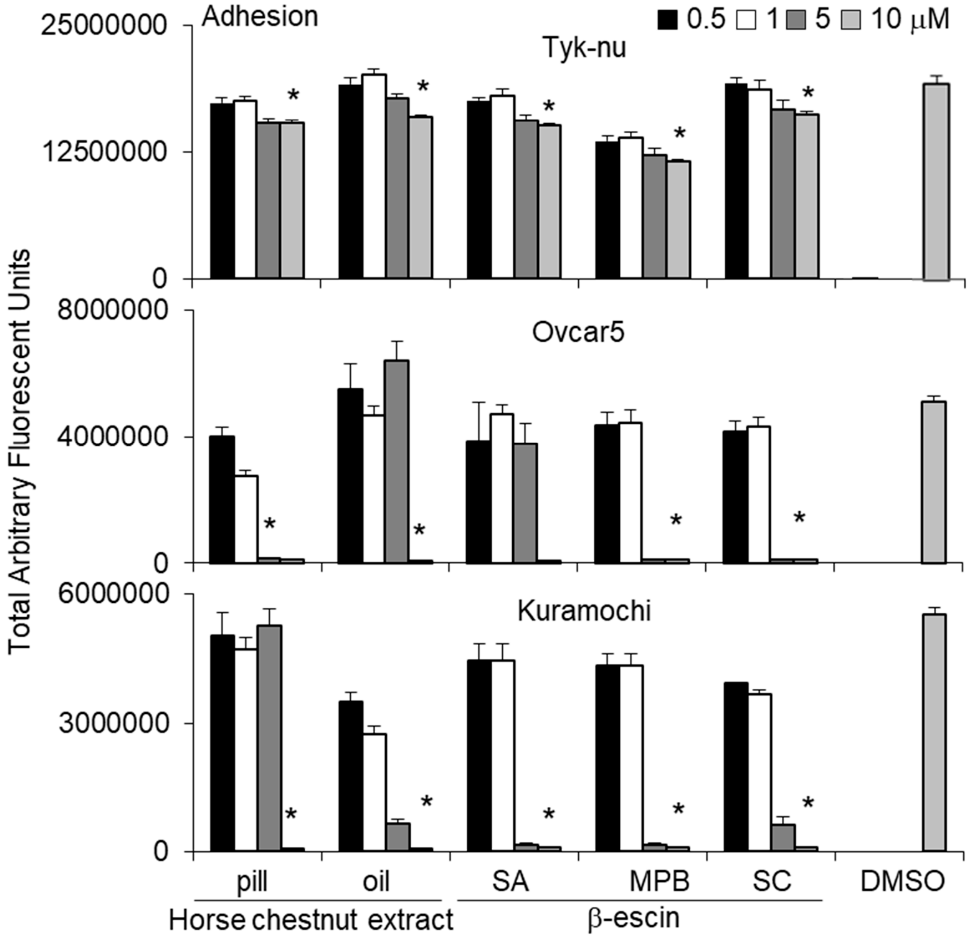

Supplementary Figure S1. Activity of different sources of β-escin on ovarian cancer cell adhesion.

Adhesion assays were performed. The effect of compounds at 4-doses were tested in three ovarian cancer (OvCa) cell lines. OvCa cell adhesion (2 hours) was tested in 384-well plates on the 3D organotypic culture. Mean+/- standard deviation. *, $p<0.05$, n=8. DMSO, dimethyl sulfoxide; SA, Sigma Aldich; MPB, MP Biomedicals; SC, Santa Cruz.

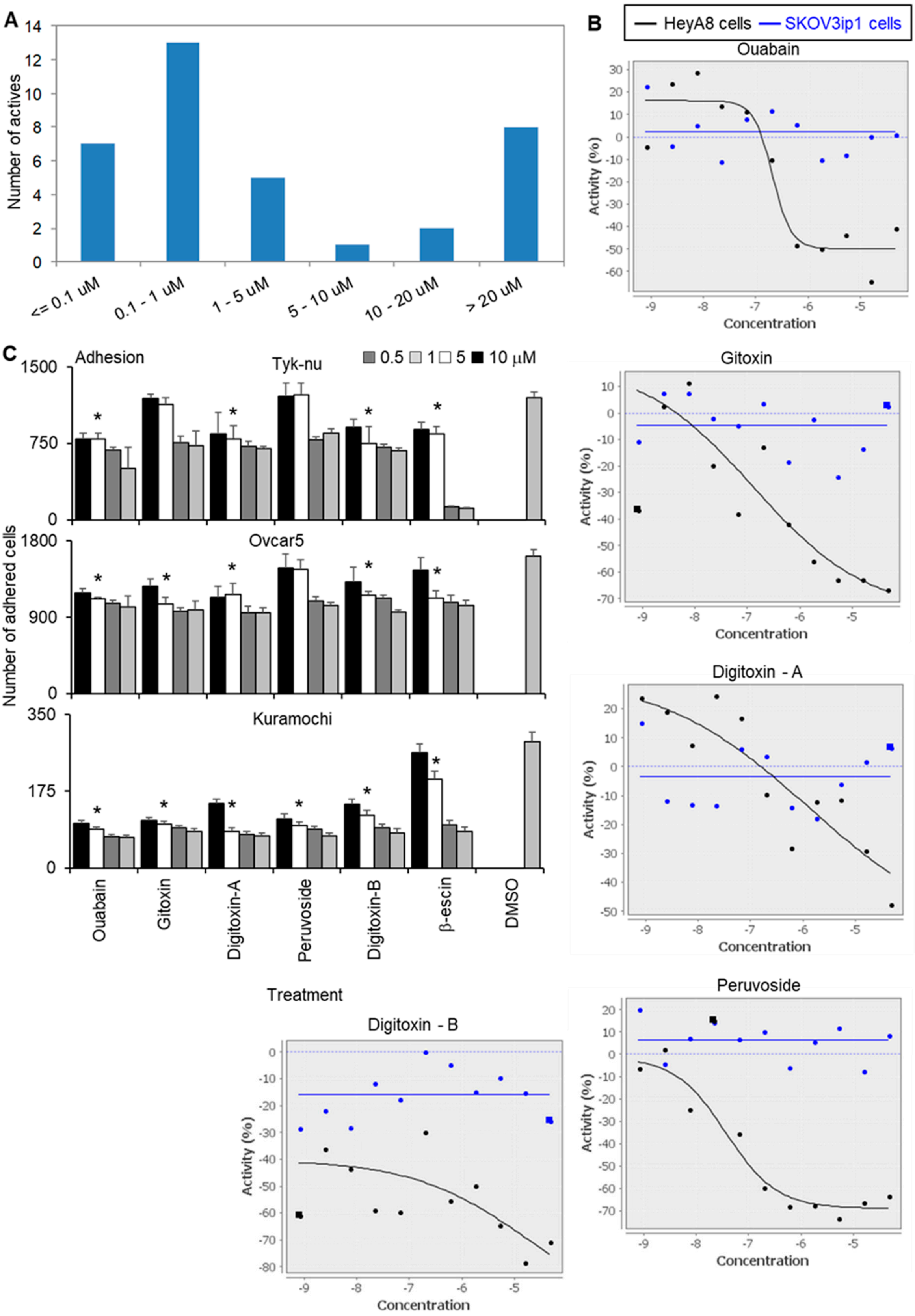

A
Number of actives
<= 0.1 uM
0.1 - 1 uM
1 - 5 uM
5 - 10 uM
10 - 20 uM
> 20 uM
B
HeyA8 cells
SKOV3ip1 cells
Ouabain
Gitoxin
Digitoxin - A
Peruvoside
Digitoxin - B
Activity (%)
Concentration
C
Adhesion
Tyk-nu
Ovcar5
Kuramochi
0.5
1
5
10 µM
Number of adhered cells
Ouabain
Gitoxin
Digitoxin-A
Peruvoside
Digitoxin-B
β-escin
DMSO
Treatment

Supplementary Figure S2. Quantitative high throughput screening with a 3D organotypic model to identify structurally similar compounds of β-escin that inhibit OvCa adhesion and invasion.

A. The number of structurally similar compounds of β-escin that were active at different doses in the primary 3D HTS adhesion/invasion assay screen.

B A confirmatory screen was performed in SKOV3ip1 and HeyA8 ovarian cancer cell lines. The 11-dose curve of the identified active compounds in 1536-well confirmatory screen are shown.

D. Secondary biological adhesion assays were performed. The effect of compounds at 4-doses were tested in three ovarian cancer (OvCa) cell lines. OvCa cell adhesion (2h) was tested in 384-well plates on the 3D organotypic culture. Mean ± standard deviation. ** $p<0.05$, $n=8$. DMSO, dimethyl sulfoxide.

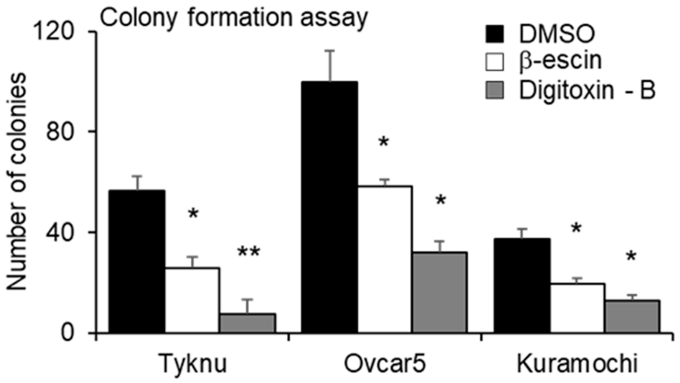

Supplementary Figure S3. Activity of β-escin and cardiac glycosides on ovarian cancer cells.

Colony formation assay showing the number of colonies formed after 10 days in Kuramochi, Ovcar5 and Tyk-nu OvCa cells treated with β-escin (10 μM) or Digitoxin (0.002 μM). Results from one of five biological repeats shown, n=2. Mean ± standard deviation. *, $p<0.05$, **, $p<0.01$.

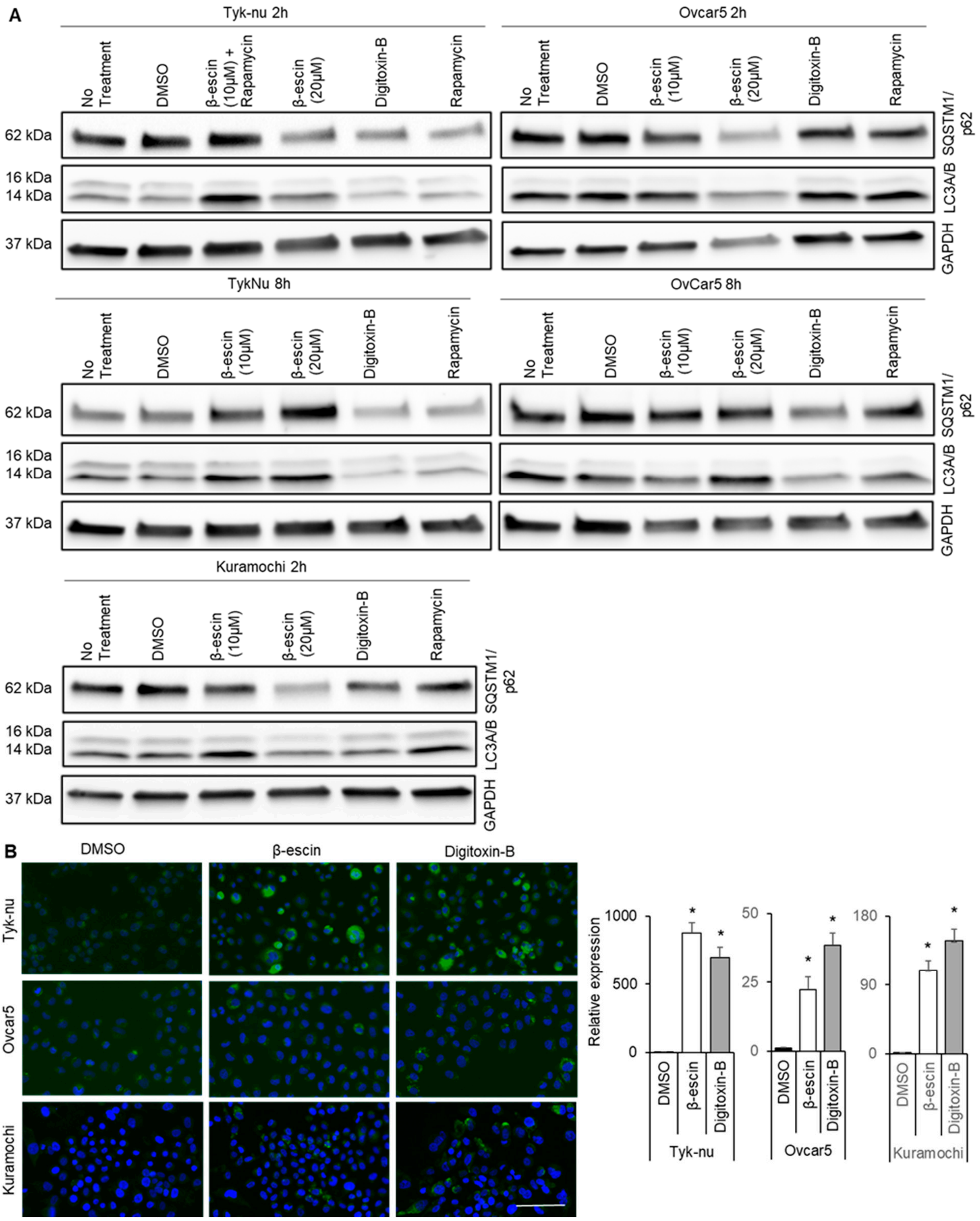
A
Tyk-nu 2h
Ovcar5 2h
No Treatment
DMSO
β-escin (10μM) + Rapamycin
β-escin (10μM)
β-escin (20μM)
Digitoxin-B
Rapamycin
62 kDa
16 kDa
14 kDa
37 kDa
SQSTM1/ p62
LC3A/B
GAPDH
TykNu 8h
OvCar5 8h
Kuramochi 2h
B
DMSO
β-escin
Digitoxin-B
Tyk-nu
Ovcar5
Kuramochi
Relative expression
1000
500
0
50
25
180
90
*

Supplementary Figure S4. β-escin regulates SQSTM1/p62 and LC3A/B production in ovarian cancer cells.

A. SQSTM1/p62 and LC3A/B expression was measured by immunoblot analysis ovarian cancer cells (OvCa) after no treatment, dimethyl sulfoxide (DMSO), β-escin (10 μM), β-escin (20 μM), Digitoxin-B (0.1μM) or Rapamycin (500nM) treatment for 2 or 8 hours.

B. OvCa cells were treatment with DMSO, β-escin (10 μM) or Digitoxin-B (0.1μM) for 8 hours and cells stained with CYTO-ID green detection reagent and Hoechst 33342 (mean ± SD, n=5). *, $p<0.05$. line, 100 μm.

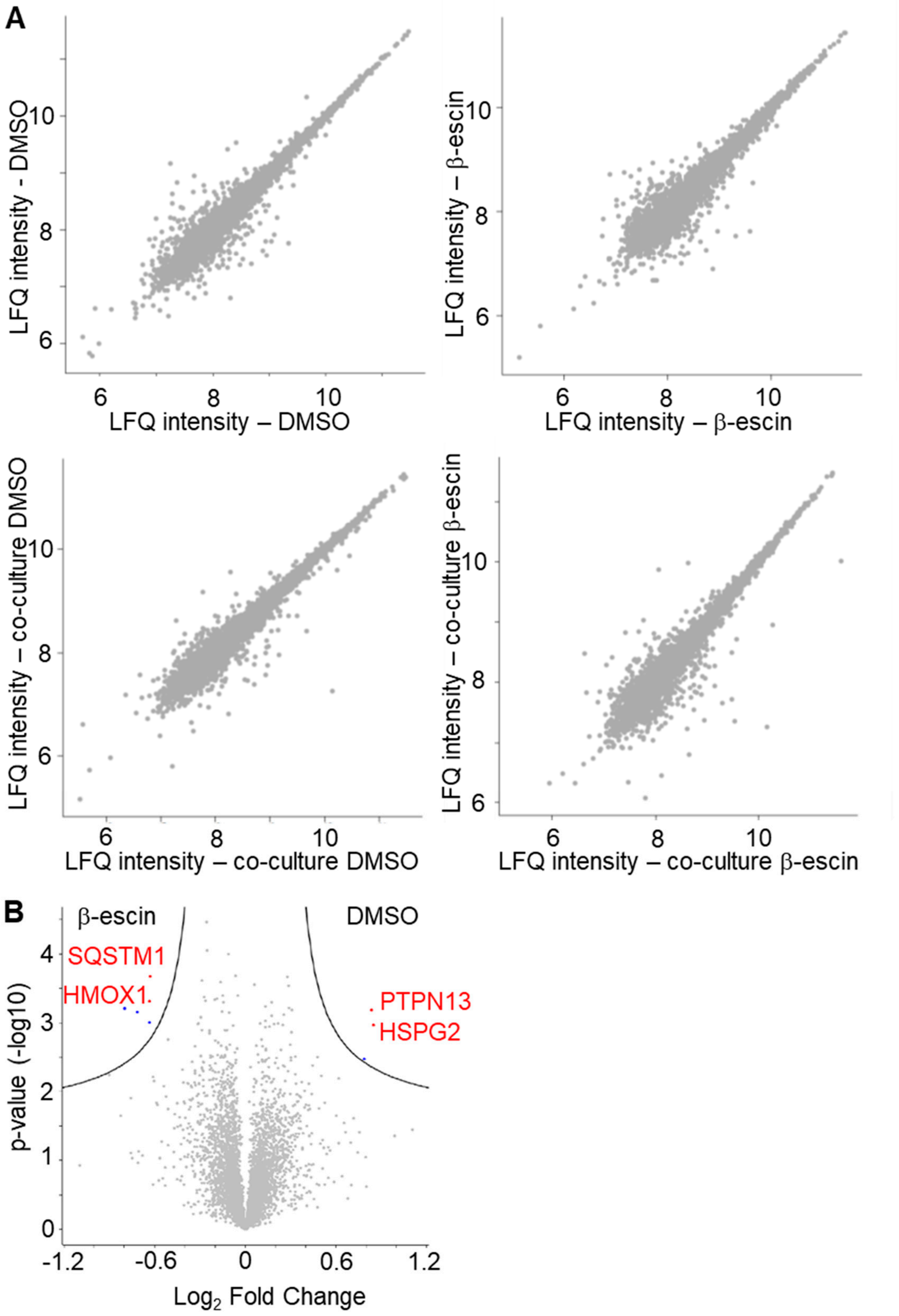
A
LFQ intensity - DMSO
LFQ intensity – DMSO
LFQ intensity – β-escin
LFQ intensity – β-escin
LFQ intensity – co-culture DMSO
LFQ intensity – co-culture DMSO
LFQ intensity – co-culture β-escin
LFQ intensity – co-culture β-escin
B
β-escin
DMSO
SQSTM1
HMOX1
PTPN13
HSPG2
p-value (-log10)
Log2 Fold Change

Supplementary Figure S5. Quality control for proteomics.

A-B. Proteomics. Mass spectrometry of proteins extracted from primary human mesothelial cells treated with DMSO, control, β-escin (5 μM) or cocultured with Tyk-nu, ovarian cancer cells, and treated with DMSO, control, or β-escin (5 μM) for 48 hours.

A. Reproducibility plots of all proteomics data, including Figure 4a-b.

B. Cell lysates were collected and measured on a Q Exactive HF mass spectrometer, and the quantification of proteins significantly altered between DMSO and β-escin treatment is shown by volcano plot (Perseus), with the significantly altered proteins highlighted in blue and red. Significance was defined by a false discovery rate (FDR) of 0.05 and an S_0 value of 0.1 (n = 4 patients per group in triplicate).

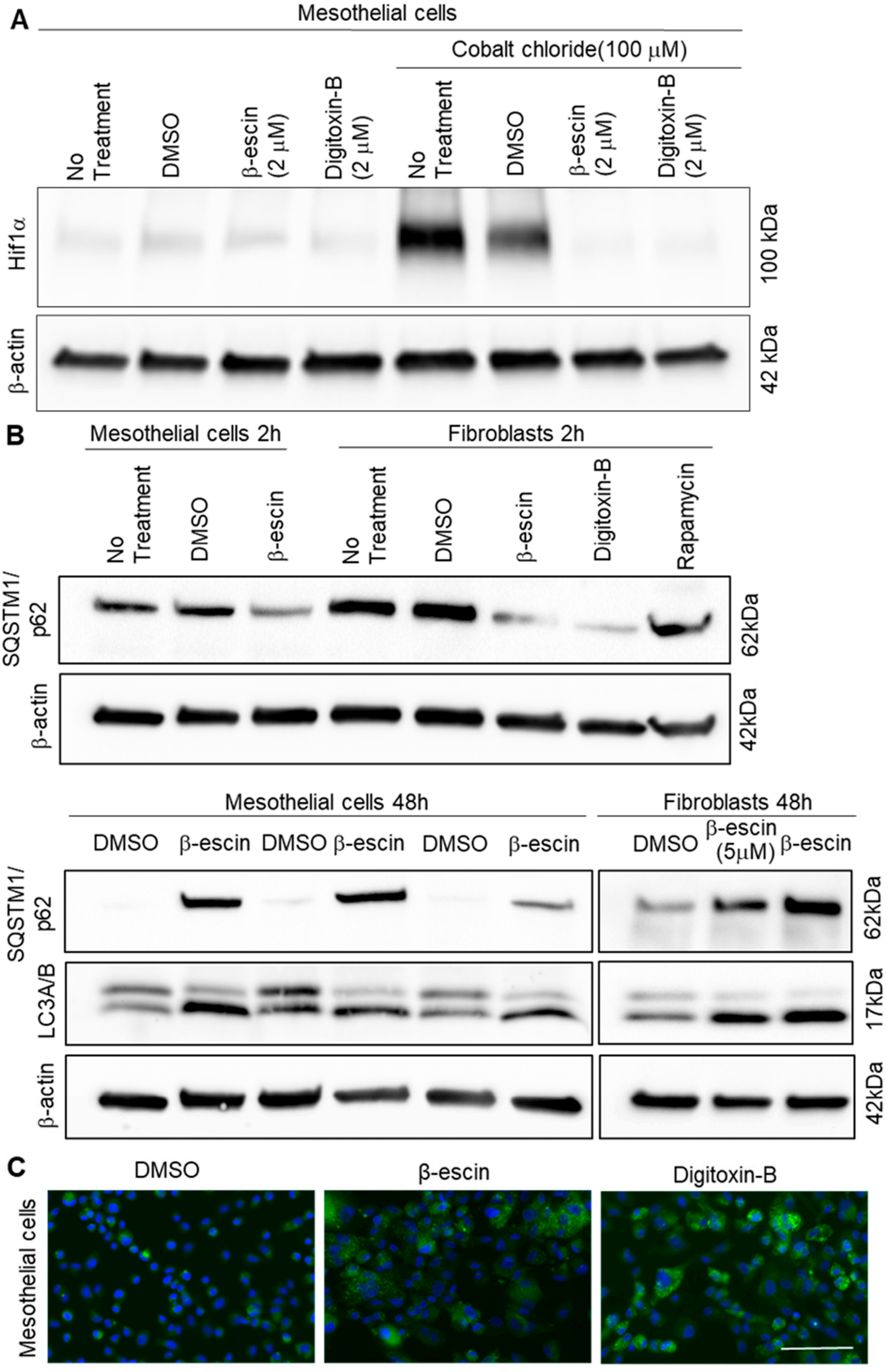
A
Mesothelial cells
Cobalt chloride(100 μM)
No Treatment
DMSO
β-escin (2 μM)
Digitoxin-B (2 μM)
No Treatment
DMSO
β-escin (2 μM)
Digitoxin-B (2 μM)
Hif1α
100 kDa
β-actin
42 kDa
B
Mesothelial cells 2h
Fibroblasts 2h
No Treatment
DMSO
β-escin
No Treatment
DMSO
β-escin
Digitoxin-B
Rapamycin
SQSTM1/ p62
62kDa
β-actin
42kDa
Mesothelial cells 48h
Fibroblasts 48h
DMSO
β-escin
DMSO
β-escin
DMSO
β-escin
DMSO
β-escin (5μM)
β-escin
SQSTM1/ p62
62kDa
LC3A/B
17kDa
β-actin
42kDa
C
DMSO
β-escin
Digitoxin-B
Mesothelial cells

Supplementary Figure S6. β-escin inhibits HIF1α production in mesothelial cells.

A. HIF1α expression was measured by immunoblot analysis in primary human mesothelial cells after no treatment, dimethyl sulfoxide (DMSO), β-escin or digitoxin in combination with or without cobalt chloride treatment for 48 hours.

B. SQSTM1/p62 expression was measured by immunoblot analysis in primary human mesothelial cells or fibroblasts after no treatment, DMSO, β-escin (10 μM), Digitoxin-B (0.1μM) or Rapamycin (500nM) treatment for 2 hours (top panel). SQSTM1/p62 and LC3A/B expression was measured by immunoblot analysis in primary human mesothelial cells or fibroblasts after DMSO, β-escin (10 μM) or β-escin (5 μM) for 48 hours (bottom panels).

C. Primary human mesothelial cells were treatment with DMSO, β-escin (10 μM) or Digitoxin-B (0.1μM) for 4 hours and cells stained with CYTO-ID green detection reagent and Hoechst 33342.

Supplemental Figure S7: Unprocessed immunoblots, densitometry reading and ratio from Figure 3C

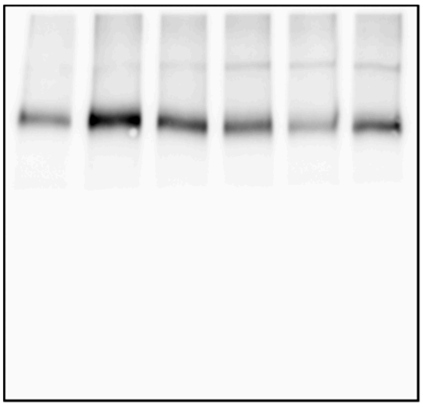

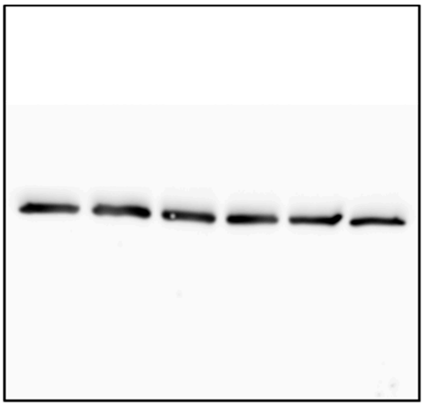

HIF-1a	b-actin	Norm.
27930	19982	1.40
63493	21561	2.94
43422	19413	2.24
34536	18597	1.86
21386	18070	1.18
36752	16800	2.19

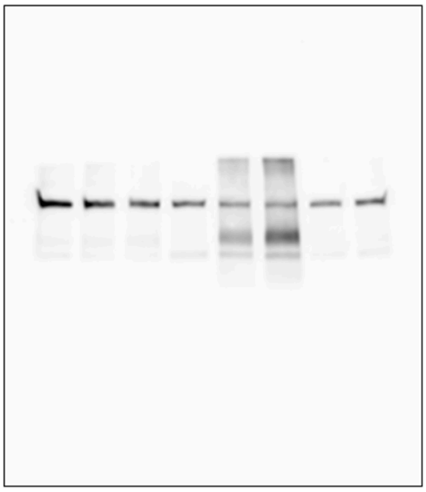

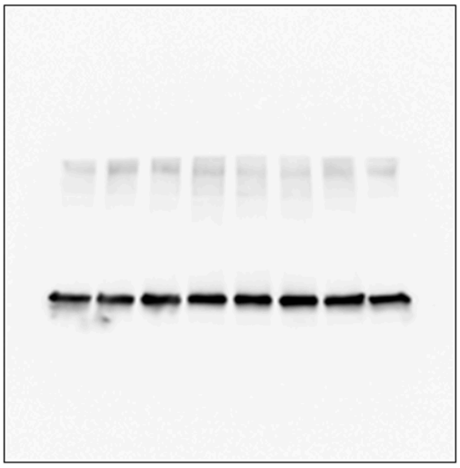

HIF1a	GAPDH	Norm.
731.8492	15961.49	1
811.3345	14622.28	0.9161
871.2635	18371.47	1.150984
6.1213	19243.21	1.205598
10249.55	19401.45	1.215518
20475.22	21110.16	1.322564
0	21418.3	1.341874
241.364	20662.47	1.294515

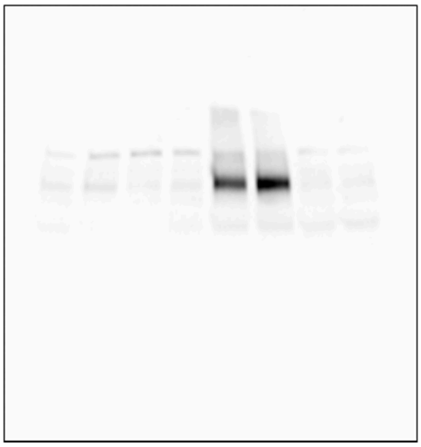

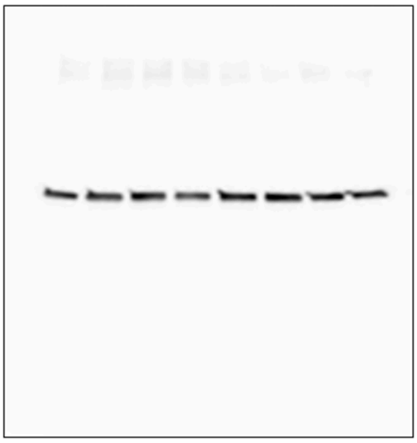

HIF1a	GAPDH	Norm.
2144.82	11640.58	1
3395.062	13929.36	1.322816
1070.021	15119.65	0.384077
1250.092	11153.92	0.608257
33156.68	15952.77	11.28025
41460.48	16730.12	13.44987
912.8492	14854.58	0.333532
484.0208	12762.46	0.205838

Supplemental Figure S8: Unprocessed immunoblots, densitometry reading and ratio from Figure 4F

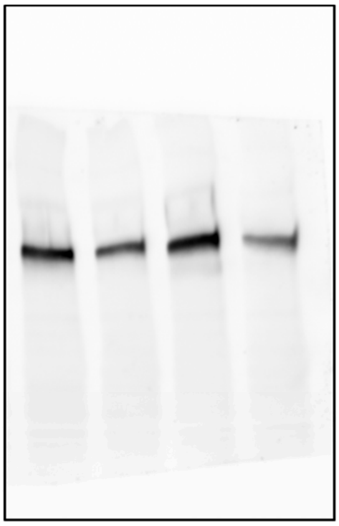

Col 1-a1	b-actin	Norm.
20887.08	13101.54	1
13310.62	12150.25	0.68716
21380.01	12515.2	1.071553
12134.04	13126.61	0.579823

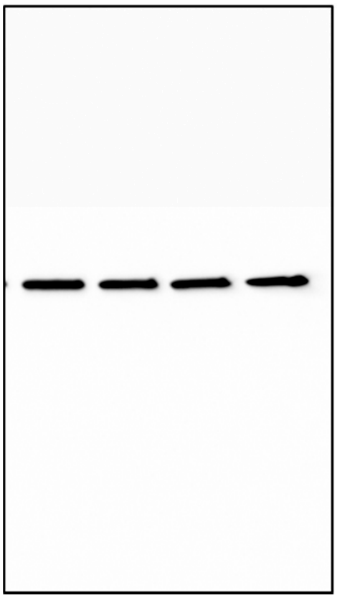

Supplemental Figure S9: Unprocessed immunoblots, densitometry reading and ratio from Figure 4G

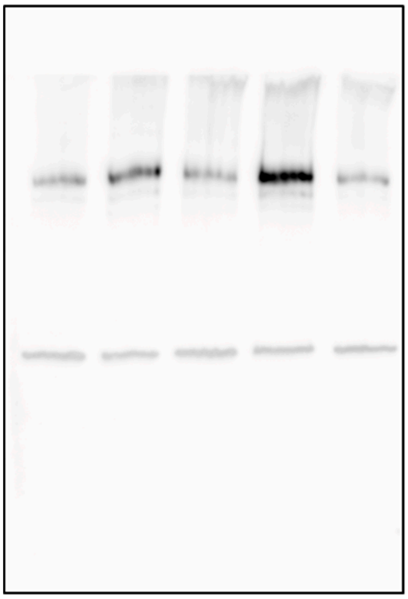

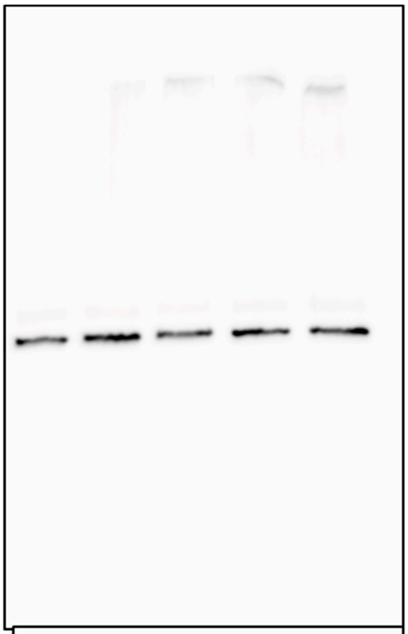

FN	b-actin	Norm.
9429.34	6441	1.46
17026.4	10017.1	1.70
13728	6617.71	2.07
32627.2	8249.13	3.96
10171.3	8433.54	1.21

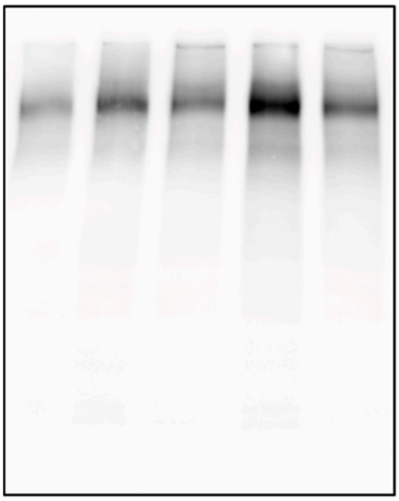

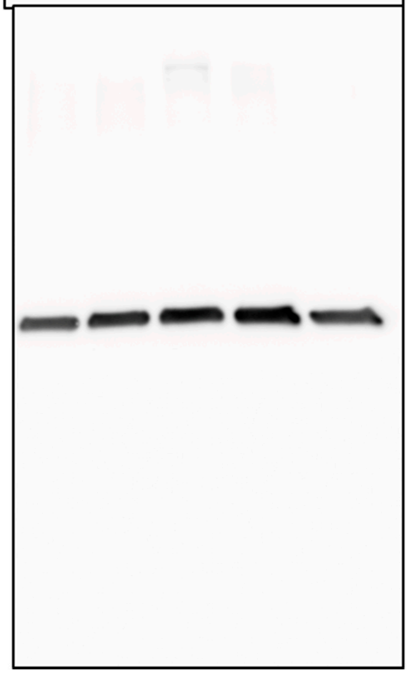

FN	b-actin	Norm.
13869	10076	1.38
28598	13028	2.20
26690	15214	1.75
52272	17729	2.95
31945	12463	2.56

Supplemental Figure S10: Unprocessed immunoblots, densitometry reading and ratio from Supplemental Figure S4A

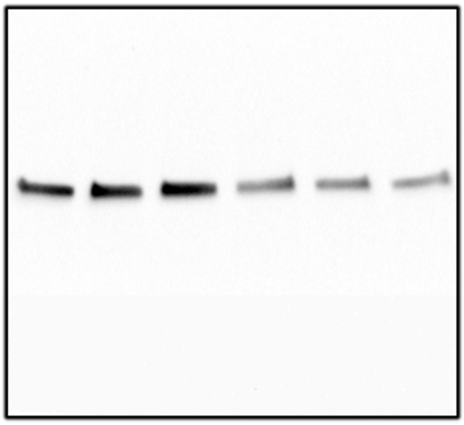

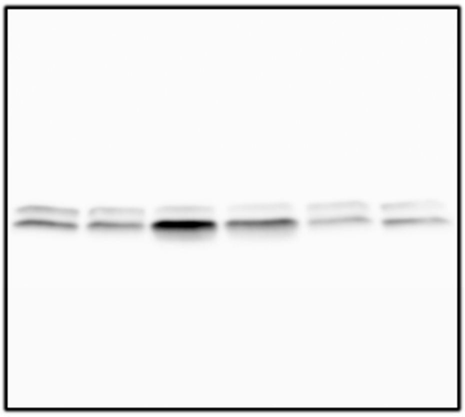

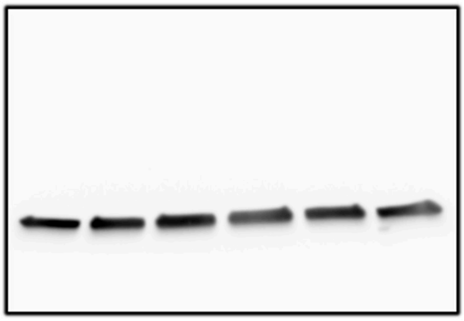

Sample	p62	GAPDH	Norm.
No Tx	14087.19	18301.6	1
DMSO	15482.48	19132.26	1.051332
B-Escin (10uM)	17663.07	20898.92	1.09801
B-Escin (20uM)	8711.459	18927.21	0.597953
Digitoxin	7063.974	18998.97	0.48304
Rapamycin	5854.681	18089.79	0.420469

Sample	LC3(A or B)	GAPDH	Ratio LCB/A	Norm. Ratio
No Tx (A)	1496.447	18301.6		
No Tx (B)	4369.196	18301.6	2.919682	1
DMSO (A)	842.7696	19132.26		
DMSO (B)	4870.853	19132.26	5.779675	1.893614
B-Escin (10uM) (A)	605.9914	20898.92		
B-Escin (10uM) (B)	12232.65	20898.92	20.18664	6.054717
B-Escin (20uM) (A)	499.9203	18927.21		
B-Escin (20uM) (B)	8448.752	18927.21	16.89987	5.596939
Digitoxin (A)	615.5772	18998.97		
Digitoxin (B)	2424.468	18998.97	3.938411	1.29941
Rapamycin (A)	692.163	18089.79		
Rapamycin (B)	2979.054	18089.79	4.304111	1.491439

Supplemental Figure S10: Unprocessed immunoblots, densitometry reading and ratio from Supplemental Figure S4A (continued)

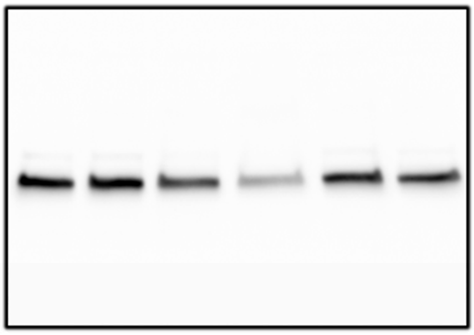

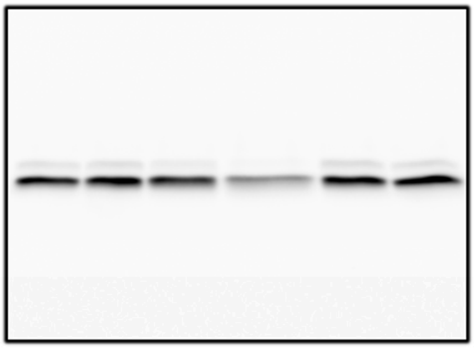

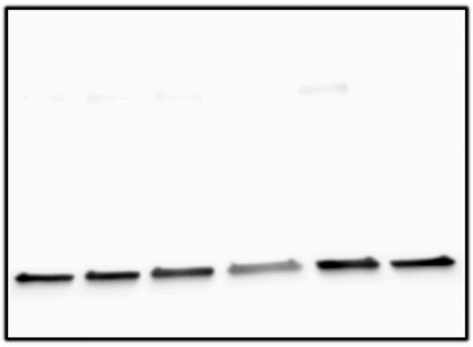

Sample	p62	GAPDH	Norm.
No Tx	19895.88	17189.28	1
DMSO	20045.1	17141.45	1.010315
B-Escin (10uM)	15935.18	18817.37	0.731634
B-Escin (20uM)	8092.811	14134.49	0.49467
Digitoxin	18232.12	23068.37	0.682838
Rapamycin	16544.9	21394.95	0.668111

Sample	LC3(A or B)	GAPDH	Ratio LC3B/A	Norm. Ratio
No Tx (A)	452.0919	17189.28		
No Tx (B)	8399.853	17189.28	18.58099	1
DMSO (A)	558.163	17141.45		
DMSO (B)	12377.92	17141.45	22.17721	1.196873
B-Escin (10uM) (A)	361.6777	18817.37		
B-Escin (10uM) (B)	11546.87	18817.37	31.92591	1.569549
B-Escin (20uM) (A)	88.9497	14134.49		
B-Escin (20uM) (B)	5909.388	14134.49	66.42124	4.347261
Digitoxin (A)	619.5772	23068.37		
Digitoxin (B)	10704.51	23068.37	17.27733	0.692866
Rapamycin (A)	398.5061	21394.95		
Rapamycin (B)	10323.22	21394.95	25.90287	1.12002

Supplemental Figure S10: Unprocessed immunoblots, densitometry reading and ratio from Supplemental Figure S4A (continued)

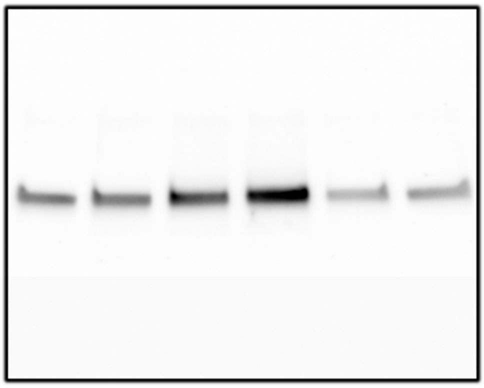

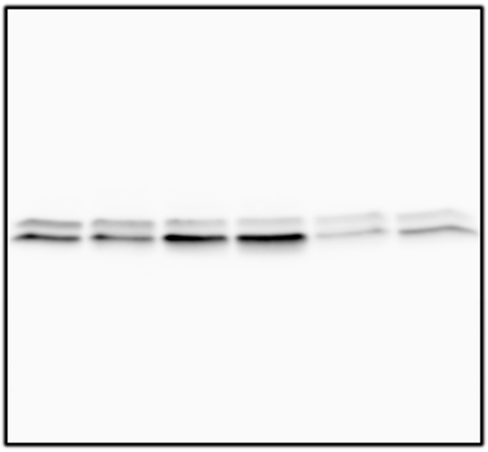

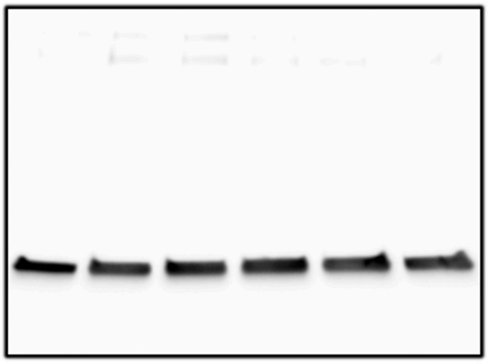

Sample	p62	GAPDH	Norm.
No Tx	34900.97	68306.52	1
DMSO	39353.72	64478.33	1.194531
B-Escin (10uM)	56357.94	71426.4	1.544262
B-Escin (20uM)	85130.84	80434.71	2.071413
Digitoxin	21337.79	75196.37	0.555362
Rapamycin	25650.43	69191.18	0.725553

Sample	LC3(A or B)	GAPDH	Ratio LC3B/LC3A	Norm. Ratio
No Tx (A)	2335.832	68306.5219	2.678192	1
No Tx (B)	6255.752	68306.5219		
DMSO (A)	1757.933	64478.3295	2.885256	1.141278
DMSO (B)	5072.045	64478.3295		
B-Escin (10uM) (A)	1300.083	71426.4005	8.551838	3.053661
B-Escin (10uM) (B)	11118.02	71426.4005		
B-Escin (20uM) (A)	947.8909	80434.7142	14.73736	4.672998
B-Escin (20uM) (B)	13969.62	80434.7142		
Digitoxin (A)	710.9914	75196.3711	2.478485	0.840639
Digitoxin (B)	1762.205	75196.3711		
Rapamycin (A)	631.5772	69191.1787	5.644311	2.080561
Rapamycin (B)	3564.782	69191.1787		

Supplemental Figure S10: Unprocessed immunoblots, densitometry reading and ratio from Supplemental Figure S4A (continued)

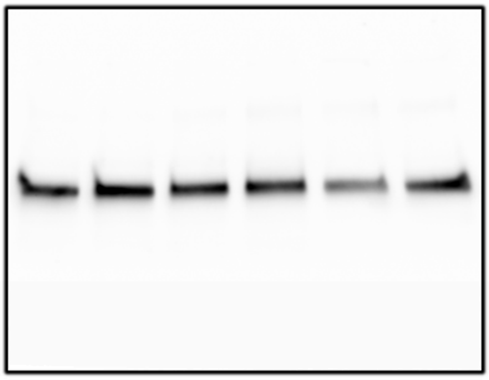

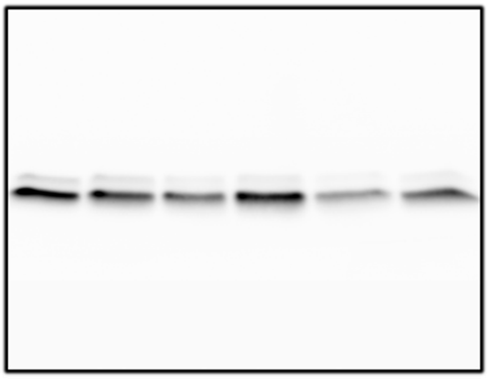

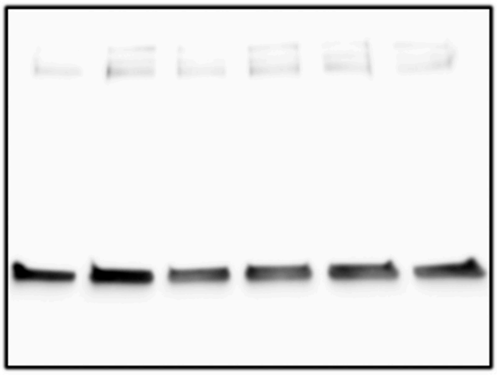

Sample	p62	GAPDH	ADJ. DENSITY
No Tx	16373.12	67791.06	1
DMSO	18372.81	82582.35	0.921148
B-Escin (10uM)	15330.64	52144.28	1.217288
B-Escin (20uM)	16284.18	61530.54	1.095758
Digitoxin	9766.154	70241.66	0.575665
Rapamycin	16246.54	71541.56	0.940249

Sample	LC3 (A or B)	GAPDH	Ratio LC3B/A	Norm. Ratio
No Tx (A)	526.234	67791.06	26.85555	1
No Tx (B)	14131.67	67791.06		
DMSO (A)	561.5772	82582.35	22.24881	0.680076
DMSO (B)	12494.38	82582.35		
B-Escin (10uM) (A)	208.6066	52144.28	45.47781	2.20155
B-Escin (10uM) (B)	9485.531	52144.28		
B-Escin (20uM) (A)	319.8492	61530.54	52.12412	2.138382
B-Escin (20uM) (B)	16673.38	61530.54		
Digitoxin (A)	148.364	70241.66	46.42418	1.668347
Digitoxin (B)	6886.167	70241.66		
Rapamycin (A)	164.7782	71541.56	65.07463	2.296098
Rapamycin (B)	10720.94	71541.56		

Supplemental Figure S10: Unprocessed immunoblots, densitometry reading and ratio from Supplemental Figure S4A (continued)

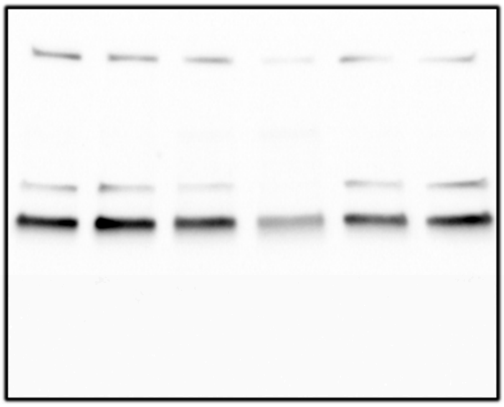

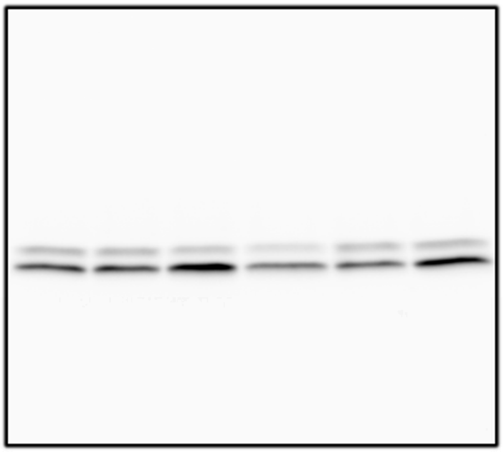

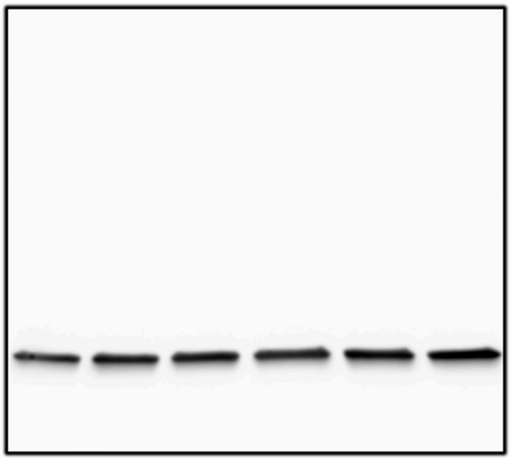

Sample	p62	GAPDH	Norm.
No Treatment	16649.35	16139.59	1
DMSO	19401.47	19246.52	0.977189
β-escin (10 μM)	15676.47	20077.23	0.756904
β-escin (20 μM)	8349.589	21907.18	0.369466
Digitoxin-B	13042.42	22388.71	0.564709
Rapamycin	16584.78	24621.42	0.65297

Sample	LC3 (A or B)	GAPDH	Ratio LC3B/A	Norm. Ratio
No Tx (A)	1778.276	16139.59		1
No Tx (B)	6030.368	16139.59	3.391095	
DMSO (A)	1909.69	19246.52		0.850962
DMSO (B)	6571.66	19246.52	3.441192	
B-Escin (10uM) (A)	1442.912	20077.23		1.684693
B-Escin (10uM) (B)	10254.56	20077.23	7.106762	
B-Escin (20uM) (A)	1091.477	21907.18		1.10614
B-Escin (20uM) (B)	5557.246	21907.18	5.09146	
Digitoxin (A)	1705.154	22388.71		0.672635
Digitoxin (B)	5395.368	22388.71	3.16414	
Rapamycin (A)	1740.397	24621.42		1.006687
Rapamycin (B)	9063.681	24621.42	5.207789	

Supplemental Figure S11: Unprocessed immunoblots, densitometry reading and ratio from Supplemental Figure S6A

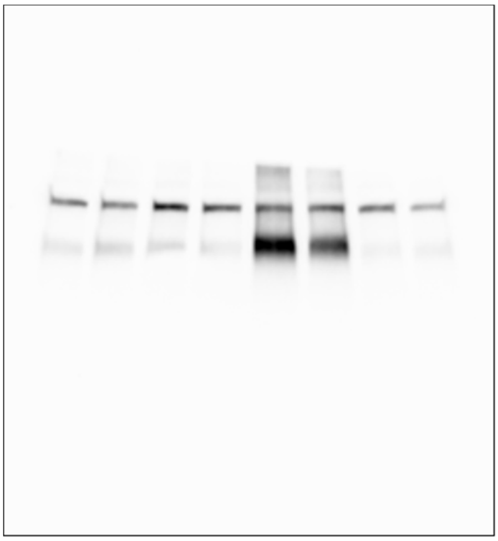

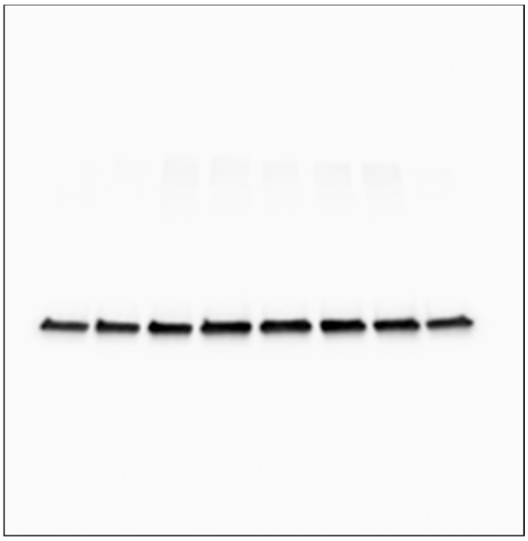

HIF1a	GAPDH	Norm.
1326.92	14856.1371	1
2267.477	15364.8442	1.652265
1740.648	17288.3797	1.127262
1213.506	21560.4005	0.630161
29509.72	21330.0366	15.48948
18204.36	21852.6432	9.326844
679.0208	18489.9863	0.411162
670.6066	15356.7939	0.488929

Supplemental Figure S12. Unprocessed immunoblots, densitometry reading and ratio from Supplemental Figure S6B

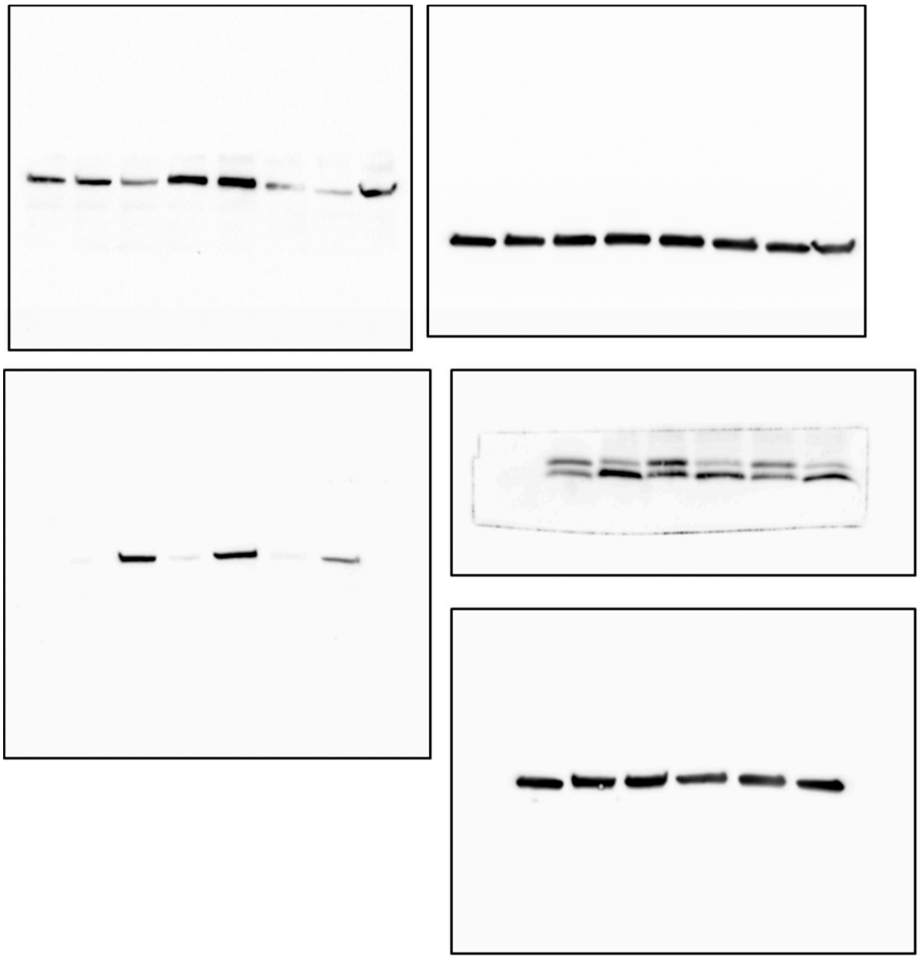

SQSTM1	b-actin	Norm.
102	20474	0.00
10489	19908	0.53
377	20357	0.02
12185	15756	0.77
299	16743	0.02
4262	19899	0.21

LC3-A/B	b-actin	Norm.
16807.69	20473.89	0.82
23169.69	19907.72	1.16
23563.64	20356.67	1.16
18492.20	15756.12	1.17
16055.44	16742.65	0.96
15665.01	19898.72	0.79

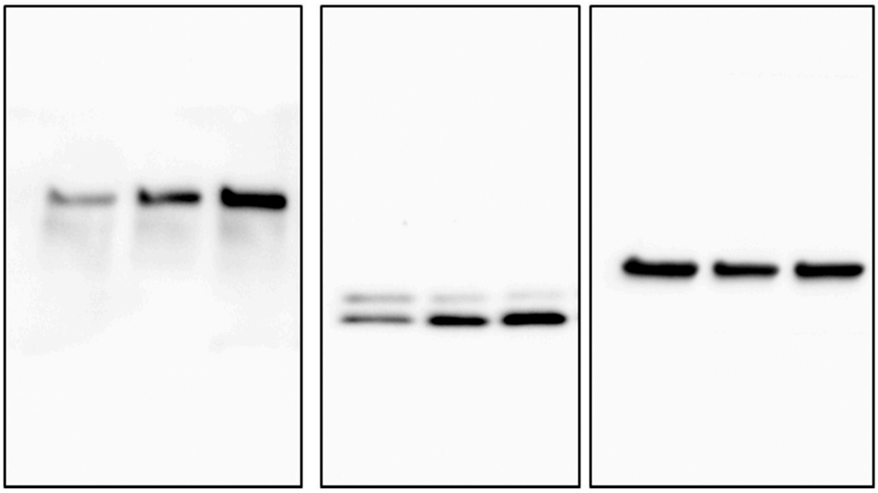

SQSTM1	b-actin	Norm.
13523	26634	0.51
18197	19202	0.95
21767	22916	0.95

LC3-A/B	b-actin	Norm.
19288.94	26633.84	0.72
28586.98	19202.11	1.49
42946.76	22915.99	1.87

Supplementary Video S1: Ovcar5 migration control/DMSO treatment.

GFP-labeled Ovcar5, ovarian cancer cells, were plated into one 96-well. The cells were treated with DMSO, control, in full growth media and the well was scratched. The video of the wound healing assay was acquired over 12 hours using an inverted fluorescent microscope with live imaging capabilities and culturing chamber.

Supplementary Video S2: Kuramochi migration control/DMSO treatment.

GFP-labeled Kuramochi, ovarian cancer cells, were plated into one 96-well. The cells were treated with DMSO, control, in full growth media and the well was scratched. The video of the wound healing assay was acquired over 18 hours using an inverted fluorescent microscope with live imaging capabilities and culturing chamber.

Supplementary Video S3: Ovcar5 migration β-escin treatment.

GFP-labeled Ovcar5, ovarian cancer cells, were plated into one 96-well. The cells were treated with β-escin 56.96 μmol/L in full growth media and the well was scratched. The video of the wound healing assay was acquired over 12 hours using an inverted fluorescent microscope with live imaging capabilities and culturing chamber.

Supplementary Video S4: Kuramochi migration β-escin treatment.

GFP-labeled Kuramochi, ovarian cancer cells, were plated into one 96-well. The cells were treated with β-escin 37.48 μmol/L in full growth media and the well was scratched. The video of the wound healing assay was acquired over 28 hours using an inverted fluorescent microscope with live imaging capabilities and culturing chamber.

Supplementary Video S5: Ovcar5 migration Digitoxin treatment.

GFP-labeled Ovcar5, ovarian cancer cells, were plated into one 96-well. The cells were treated with Digitoxin 0.96 μmol/L in full growth media and the well was scratched. The video of the wound healing assay was acquired over 12 hours using an inverted fluorescent microscope with live imaging capabilities and culturing chamber.

Supplementary Video S6: Kuramochi migration Digitoxin treatment.

GFP-labeled Tyk-nu, ovarian cancer cells, were plated into one 96-well. The cells were treated with Digitoxin 0.96 μmol/L in full growth media and the well was scratched. The video of the wound healing assay was acquired over 28 hours using an inverted fluorescent microscope with live imaging capabilities and culturing chamber.

References for Supplementary Information

1 Hart, P. C. *et al.* Mesothelial Cell HIF1alpha Expression Is Metabolically Downregulated by Metformin to Prevent Oncogenic Tumor-Stromal Crosstalk. *Cell reports* **29**, 4086-4098 e4086, doi:10.1016/j.celrep.2019.11.079 (2019).
